# Therapeutic Helminth Infection of Macaques with Idiopathic Chronic Diarrhea Alters the Inflammatory Signature and Mucosal Microbiota of the Colon

**DOI:** 10.1371/journal.ppat.1003000

**Published:** 2012-11-15

**Authors:** Mara Jana Broadhurst, Amir Ardeshir, Bittoo Kanwar, Julie Mirpuri, Uma Mahesh Gundra, Jacqueline M. Leung, Kirsten E. Wiens, Ivan Vujkovic-Cvijin, Charlie C. Kim, Felix Yarovinsky, Nicholas W. Lerche, Joseph M. McCune, P'ng Loke

**Affiliations:** 1 Division of Experimental Medicine, Department of Medicine, University of California San Francisco, San Francisco, California, United States of America; 2 California National Primate Research Center, Davis, California, United States of America; 3 Department of Immunology, University of Texas Southwestern Medical Center, Dallas, Texas, United States of America; 4 Department of Microbiology, New York University, New York, New York, United States of America; National Institutes of Health, National Institute of Allergy and Infectious Diseases, United States of America

## Abstract

Idiopathic chronic diarrhea (ICD) is a leading cause of morbidity amongst rhesus monkeys kept in captivity. Here, we show that exposure of affected animals to the whipworm *Trichuris trichiura* led to clinical improvement in fecal consistency, accompanied by weight gain, in four out of the five treated monkeys. By flow cytometry analysis of pinch biopsies collected during colonoscopies before and after treatment, we found an induction of a mucosal T_H_2 response following helminth treatment that was associated with a decrease in activated CD4^+^ Ki67+ cells. In parallel, expression profiling with oligonucleotide microarrays and real-time PCR analysis revealed reductions in T_H_1-type inflammatory gene expression and increased expression of genes associated with IgE signaling, mast cell activation, eosinophil recruitment, alternative activation of macrophages, and worm expulsion. By quantifying bacterial 16S rRNA in pinch biopsies using real-time PCR analysis, we found reduced bacterial attachment to the intestinal mucosa post-treatment. Finally, deep sequencing of bacterial 16S rRNA revealed changes to the composition of microbial communities attached to the intestinal mucosa following helminth treatment. Thus, the genus *Streptophyta* of the phylum Cyanobacteria was vastly increased in abundance in three out of five ICD monkeys relative to healthy controls, but was reduced to control levels post-treatment; by contrast, the phylum Tenericutes was expanded post-treatment. These findings suggest that helminth treatment in primates can ameliorate colitis by restoring mucosal barrier functions and reducing overall bacterial attachment, and also by altering the communities of attached bacteria. These results also define ICD in monkeys as a tractable preclinical model for ulcerative colitis in which these effects can be further investigated.

## Author Summary

Young macaques kept in captivity at Primate Research Centers often develop chronic diarrhea, which is difficult to treat because it is poorly understood. This disease shares many features with ulcerative colitis, which is an autoimmune disease affecting the intestinal tract of humans. Recently, parasitic worms have been used in clinical trials to treat inflammatory bowel diseases in humans with positive results, but very little is known about how worms can improve symptoms. We performed a trial where we treated macaques suffering from chronic diarrhea with human whipworms, collecting gut biopsies before and after treatment. We found that 4 out of the 5 treated macaques improved their symptoms and studied the changes in their gut immune responses, as they got better. We found that after treatment with worms, the monkeys had less bacteria attached to their intestinal wall and a reduced inflammatory response to the gut bacteria. Additionally, the composition of gut bacteria was altered in the sick macaques and was restored close to normal after treatment with whipworms. These results provide a potential mechanism by which parasitic worms may improve the symptoms of intestinal inflammation, by reducing the immune response against intestinal bacteria.

## Introduction

The incidence of inflammatory bowel diseases (IBD) is highest in industrialized regions wherein helminth infections have been largely eliminated, raising the hypothesis that helminths may protect against intestinal inflammation underlying the disease [1]. Indeed, there is evidence that experimental helminth treatment can ameliorate symptoms in IBD patients [2], [3] and in mice [4], [5], [6], [7]. This effect may be attributable to the induction of immunoregulatory networks [8], [9] and mucosal repair by a T_H_2-type immune response [10], [11]. The pig whipworm, *T. suis*, is currently being used in clinical trials to alleviate symptoms in IBD patients, but the mucosal immune responses in treated patients has not been investigated [11].

Recently, we characterized the mucosal responses of an ulcerative colitis (UC) patient who self-infected with the human parasite *T. trichiura* [12]. These studies led to the proposal that the T_H_2 immune response activated for parasite clearance, as well as the induction of IL-22 expression, may promote mucosal healing in UC patients by increasing mucus production and turnover of intestinal epithelial cells [11]. In a different study, the mucosal responses of celiac disease patients were examined in detail after experimental treatment with human hookworm infection, showing a suppression of T_H_17 responses and upregulation of IL-22 [13], [14], [15]. This growing body of clinical evidence suggests that a better understanding of mucosal responses activated by helminth infections may contribute to new IBD therapies. Non-rodent, preclinical models of IBD are lacking, and would greatly benefit the development of such novel therapeutics.

ICD, leading to progressive weight loss and dehydration [16], frequently afflicts captive juvenile rhesus macaques (*Macaca mulatta*) and is a leading cause of death at primate research centers. The clinical management of these animals is difficult because the condition is often refractory to available treatment. Intestinal inflammatory pathology during ICD can be similar to that found in UC and it has been suggested that ICD may be an informative model for UC [17], [18]. As in the case of UC, inflammation is more severe in the colon than in the jejunum and it has been shown that dysregulation of IL-6-STAT3 activation may be an important inflammatory mediator [19]. In this study, we sought to characterize the mucosal inflammatory response driving ICD in the rhesus monkey, and to determine whether helminth exposure can modulate this inflammatory response and lead to clinical improvement.

It is conceivable that the protective effects of helminth treatment for IBD patients may be partly attributable to alterations in the microbial communities of the intestinal tract [11], [20]. Changes to the intestinal mucosa by helminth infection [21] are likely to have a major impact on the gut microbial environment [11]. *H. polygyrus* infection has been shown to have major effects on the microbiota of mice, especially increasing the abundance of the Lactobacillaceae family [20]. Colonization of the colon with *T. muris* has been shown to be dependent on the gut microbiota [22]. Uncultured bacterial communities can now be analyzed using DNA barcoding and deep sequencing approaches to investigate how interactions between communities can influence disease pathogenesis [23], [24]. In a previous cross-sectional study, McKenna *et al.* investigated the microbiome of healthy macaques in comparison to macaques with chronic diarrhea and after SIV infection [25], finding that bacterial diversity was significantly lower and the family Campylobacteraceae was more common in monkeys with colitis. In this study, we have measured the quantity of attached bacteria to the intestinal mucosa pre-and post-helminth treatment, and further characterized the composition of bacterial communities by deep sequencing in order to determine whether helminth treatment alters the communities of attached bacteria within the intestinal tract of macaques with ICD.

## Results

### Clinical responses to *T. trichiura* ova

**Figure 1:**
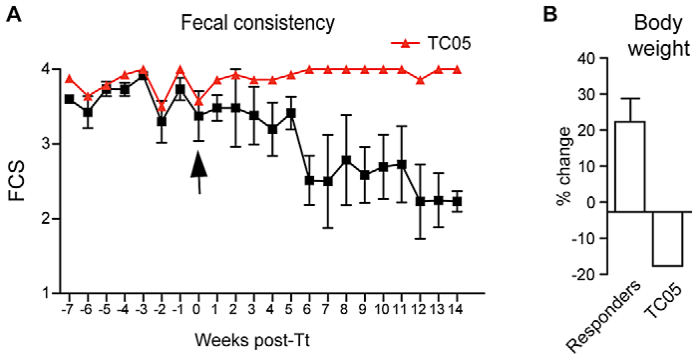
Clinical improvement of juvenile macaques with chronic diarrhea following *T. trichiura* treatment. (**A**) Diarrhea of five macaques was scored daily before and after oral administration of germinated *T. trichiura* (Tt) ova. Fecal consistency scores (FCS) were based on a standardized 4-point scale; 1 = Well-formed, normal; 1.5 = Normal to semi-solid; 2 = Semi-solid to normal; 2.5 = Semi-solid; 3 = Semi-solid to liquid; 3.5 = Liquid to semi-solid; 4 = Liquid. Semi solid stool is defined as “porridge-like” or able to be picked up with a fork. Each score represents the average of daily scores per week. Subject TC05 (red line) showed no clinical response while four other responder animals (TR01-TR04; black line) had a decrease in FCS. (**B**) Change in body weight at 14 weeks following *T. trichiura* treatment, expressed as a percentage of body weight at the time of egg administration. Tt refers to *T. trichiura* treatment.

Five juvenile rhesus macaques diagnosed with ICD were enrolled (see Methods for selection criteria) and monitored daily before and after oral administration of 1000 *T. trichiura* ova (Figure 1). The extent of diarrhea (Figure 1) was scored daily and an improvement in fecal consistency (Figure 1A), accompanied by weight gain (Figure 1B), was observed in four out of the five monkeys (TC01–TC04) following *T. trichiura* treatment. Subject TC05 continued to have severe diarrhea and weight loss, and eventually had to be euthanized. Importantly, patent infection was not established in any of the monkeys despite symptomatic improvements and eggs were never detected in the feces. Spontaneous remission does not typically occur in untreated animals, which maintain similar fecal consistency scores in other experiments (Figure S1).

### Flow cytometry analyses of leukocytes from pinch biopsies show increased mucosal T_H_2 responses and reduced immune activation

**Figure 2:**
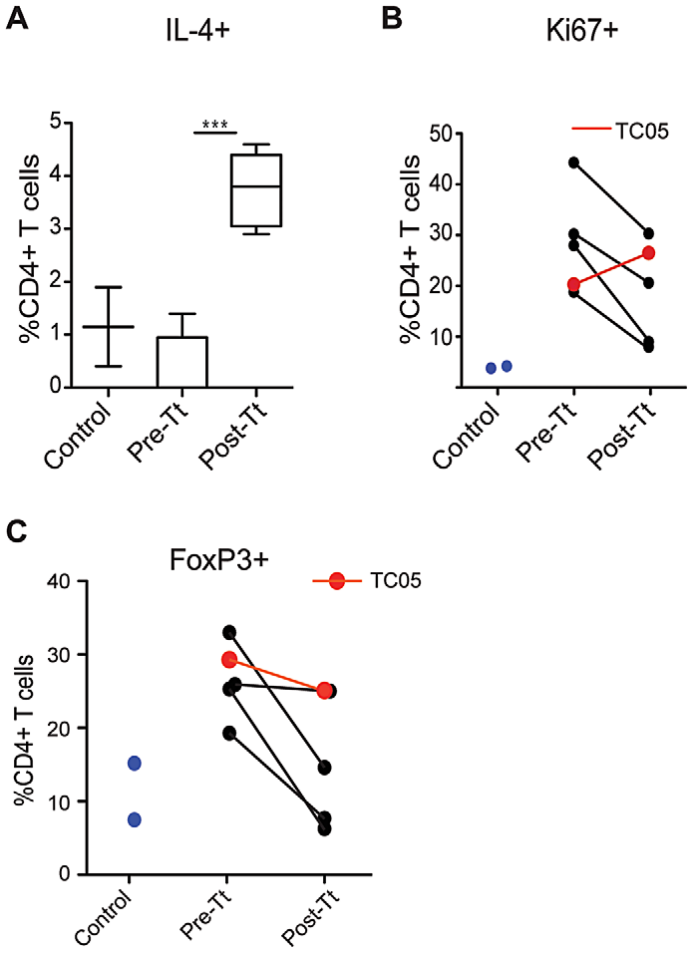
Flow cytometric analysis of CD4^+^ T cells from colon biopsies following *T. trichiura* treatment. Pinch biopsies collected during colonoscopies pre-treatment (Pre-Tt) and 14 weeks post-treatment (Post-Tt) were compared with samples collected from healthy age-matched controls (Control). Lamina propria mononuclear cells (LPMCs) from biopsies were stimulated *ex vivo* with PMA and ionomycin for intracellular cytokine staining. (**A**) Box and whisker plot showing the percentage of IL-4+ cells among CD4+ LPMCs. ***p<0.001, determined by Student's *t*-test. LPMCs were also stained *ex vivo* for intranuclear (**B**) Ki67 and (**C**) FoxP3. Lines connect paired samples from the same subjects pre- and post-treatment. The non-responding macaque (TC05) is shown in red.

To assess changes to the intestinal mucosa before and after helminth treatment, colonoscopies were performed and mucosal biopsies were collected from the five animals prior to treatment, as well as from two healthy, age-matched controls. A second colonoscopy was performed on the ICD subjects 14 weeks after oral administration of *T. trichiura* ova. Peripheral blood mononuclear cells (PBMC) and lamina propria mononuclear cells (LPMC) harvested from colon biopsies were stimulated with PMA and ionomycin for intracellular cytokine staining and flow cytometry analysis (FACS). The proportion of colonic CD4^+^ T cells producing IL-4, but not those producing IFNγ, was significantly higher (p<0.001) following *T. trichiura* treatment (Figure 2A), revealing a localized T_H_2 response. In PBMCs, we did not detect more CD4^+^ T cells producing IL-4 after treatment (data not shown). All five ICD subjects demonstrated this response, indicating that the lack of clinical improvement in subject TC05 was not due to the absence of a T_H_2 response. The colonic mucosa of ICD subjects also showed a higher proportion of Ki67^+^ CD4^+^ T cells (Figure 2B), indicative of ongoing inflammation. The four clinical responders showed a diminished fraction of such cells following *T. trichiura* treatment, while their proportion actually increased in subject TC05. The proportion of FoxP3^+^ CD4^+^ regulatory T (T_reg_) cells was greater in the colonic mucosa of the ICD subjects compared to healthy controls and was markedly reduced in three out of four clinical responders following *T. trichiura* treatment (Figure 2C). This observation suggests that the local expansion of T_regs_ in these subjects was reflective of active ongoing inflammation.

### Transcriptional profiling analysis demonstrates alterations to inflammatory gene expression patterns in response to *T. trichiura* treatment

**Figure 3:**
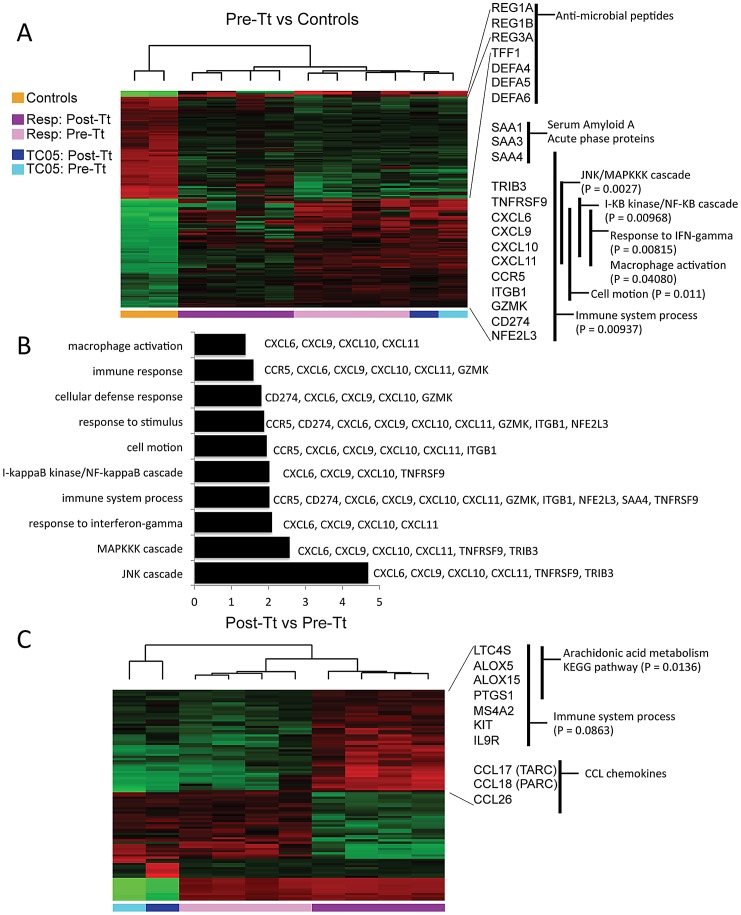
Transcriptional profiling of colon biopsies by macaque whole genome oligonucleotide microarrays. RNA was isolated from pinch biopsies collected during colonoscopies for microarray analysis. Hierarchical clustering analysis of samples and genes based on the expression levels of genes differentially expressed (B>0) between (**A**) pre-treatment colitis samples and healthy controls, where each column represents an individual subject and each row represents a single gene. Black indicates median levels of expression; red, greater than median expression; green, less than median expression. Colored horizontal bars indicate the clustering of samples collected from healthy controls (Controls, orange), clinical responders [pre-treatment (Resp: Pre-Tt, light purple) and post-treatment (Resp: Post-Tt, dark purple)], and subject TC05 [pre-treatment (TC05: Pre-Tt, light blue) and post-treatment (TC05: Post-Tt, dark blue)]. Representative genes are listed in the biological processes that are overrepresented. (**B**) GO analyses identified biological processes up-regulated in pre-treatment colitis samples. *X* axis indicates the amount of statistical significance [as −log(*P*)] in enrichment for the indicated biological process, with the up-regulated genes in this process listed. (**C**) Hierarchical clustering analysis of samples and genes based on the expression levels of genes differentially expressed (B>0) between colitis samples before and after *T. trichiura* treatment. The complete list of genes for (A) is shown in Figure S2 and Table S1. The complete list of genes for (C) is shown in Figure S3 and Table S2.

We isolated RNA from pinch biopsies paired with the samples used for FACS analysis. Whole genome expression profiling of colon biopsy samples identified 185 transcripts that distinguished the inflamed mucosa of ICD subjects (prior to *T. trichiura* treatment) from healthy colon tissue (Figure 3A, Figure S2, Table S1). The genes upregulated in ICD samples included classical T_H_1-type inflammatory mediators such as inducible nitic oxide synthase (NOS2), chemokines (CXCL9, CXCL10, CXCL11), and serum amyloid A (SAA1, SAA3, SAA4). IBD-associated genes implicated in mucosal injury and defense [including regenerative factors (REG1, REG3), trefoil peptides (TFF1), and defensins (MNP2, ROAD1, ROAD2)] were also upregulated in ICD samples. This transcriptional signature is consistent with previous studies on UC patients [12], [26] and could be driven by attached mucosal microbiota [26]. Pathway analysis indicates enrichment for genes in the biological processes that include the JNK cascade, MAPKKK cascade, response to IFN-g, I**κ**B Kinase/NF-**κ**b cascade and macrophage activation as shown in Figure 3B.

**Figure 4:**
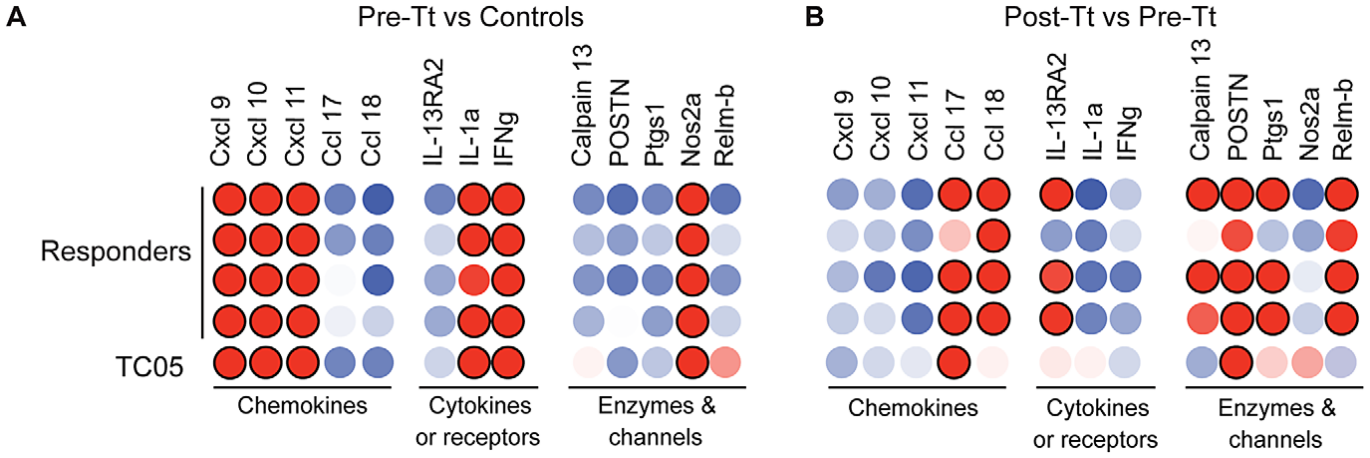
Real-time PCR verification of genes differentially expressed between groups. (**A**) Expression levels of genes in pre-treatment (Pre-Tt) colitis subjects are shown relative to the average expression of samples from healthy controls. Red/pink spots denote higher expression and blue spots represent lower expression; differential expression increases with spot intensity. Red spots with black borders represent >10-fold higher expression. (**B**) Expression levels of genes for samples taken post-treatment (Post-Tt) for each subject are shown relative to pre-treatment levels from the same subject.

Changes in gene expression induced by helminth treatment were also evaluated and 99 transcripts were found to be significantly altered following *T. trichiura* treatment (Figure 3B, Figure S3, Table S2). Notably, many of the IBD-associated genes identified in ICD samples were downregulated following *T. trichiura* treatment. Furthermore, post-treatment samples demonstrated the induction of T_H_2-type immune response pathways, including those associated with IgE signaling (FCER1A, MS4A2), mast cell activation (CPA3, CMA1), T_H_2 and eosinophil recruitment (CCL17, CCL18, CCL26), alternative activation of macrophages (ALOX5, ALOX15), cytokine signaling (IL5RA, IL9R, POSTN), and worm expulsion (RELMB). This shift was confirmed by quantitative reverse transcription (qRT)-PCR (Figure 4). KEGG pathway analysis showed significant (P = 0.0136) enrichment for genes in the arachidonic acid pathway (ALOX5, ALOX15, LTC4S, PTGS1) indicating the production of prostaglandins, which are important regulators of the inflammatory response [27].

The transcriptional profile of subject TC05 clustered separately from the four clinical responders, a distinction primarily driven by a group of immunoglobulin-related transcripts present at much lower levels in this subject (Figure S4 and Table S3). TC05 did not demonstrate a shift from a T_H_1-type to T_H_2-type gene expression pattern following *T. trichiura* treatment as seen in the four responders, resulting in the close hierarchical clustering between pre- and post-treatment samples from this animal. This observation was confirmed by qRT-PCR (Figure 4), as TC05 did not show reduced expression of certain type-1 inflammatory genes (NOS2, IL1A) or increased expression of T_H_2-type response genes (CCL18, CALPAIN13, RELMB) following *T. trichiura* treatment.

### Increased bacterial attachment to the intestinal mucosa during ICD

**Figure 5:**
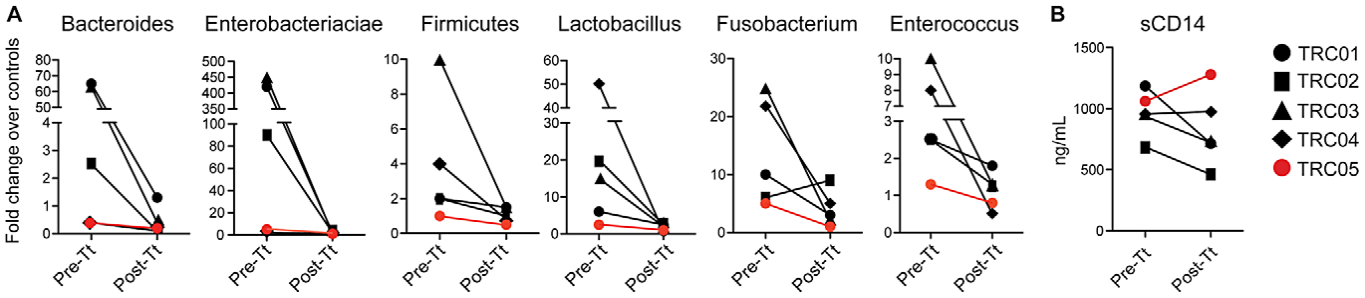
*T. trichiura* treatment reduces bacterial attachment to the colonic mucosa. (**A**) DNA samples harvested from colon biopsies taken pre-treatment (Pre-Tt) and post-treatment (Post-Tt) were analyzed by quantitative PCR for the abundance of bacterial taxa based on specific primers for 16S rRNA sequences. Abundance is expressed as a fold-change above the average value of healthy controls. Subject TC05 is shown in red. (**B**) Plasma levels of soluble CD14 were measured by ELISA.

Since IBD may be driven by an aberrant immune response against commensal gut bacteria due to changes in bacterial attachment to the intestinal mucosa [28], [29], we quantified the abundance of several bacterial taxa in colon biopsy samples using quantitative PCR for 16S ribosomal RNA genes. Biopsies from each ICD subject demonstrated a higher abundance of multiple taxa compared to healthy controls, revealing a broad increase in bacterial attachment (Figure 5A). Bacterial attachment was reduced following *T. trichiura* treatment, suggesting that the defective mucosal barrier was partially restored. Surprisingly, the bacterial loads in biopsies from TC05 were only slightly higher than those found in control biopsies and only slightly reduced post treatment, suggesting that increased bacterial attachment may not have driven colitis in this subject. One possibility would be that subject TC05 is more sensitive to bacterial translocation even with small quantities of attached bacteria because of an inherently leaky mucosal barrier. Consistent with this possibility, only TC05 showed an increase in serum soluble CD14 (a correlate of microbial product translocation across the mucosal barrier [30]) following *T. trichiura* treatment (Figure 5B).

### Changes to the composition of attached bacteria in response to *T. trichiura* ova

**Figure 6:**
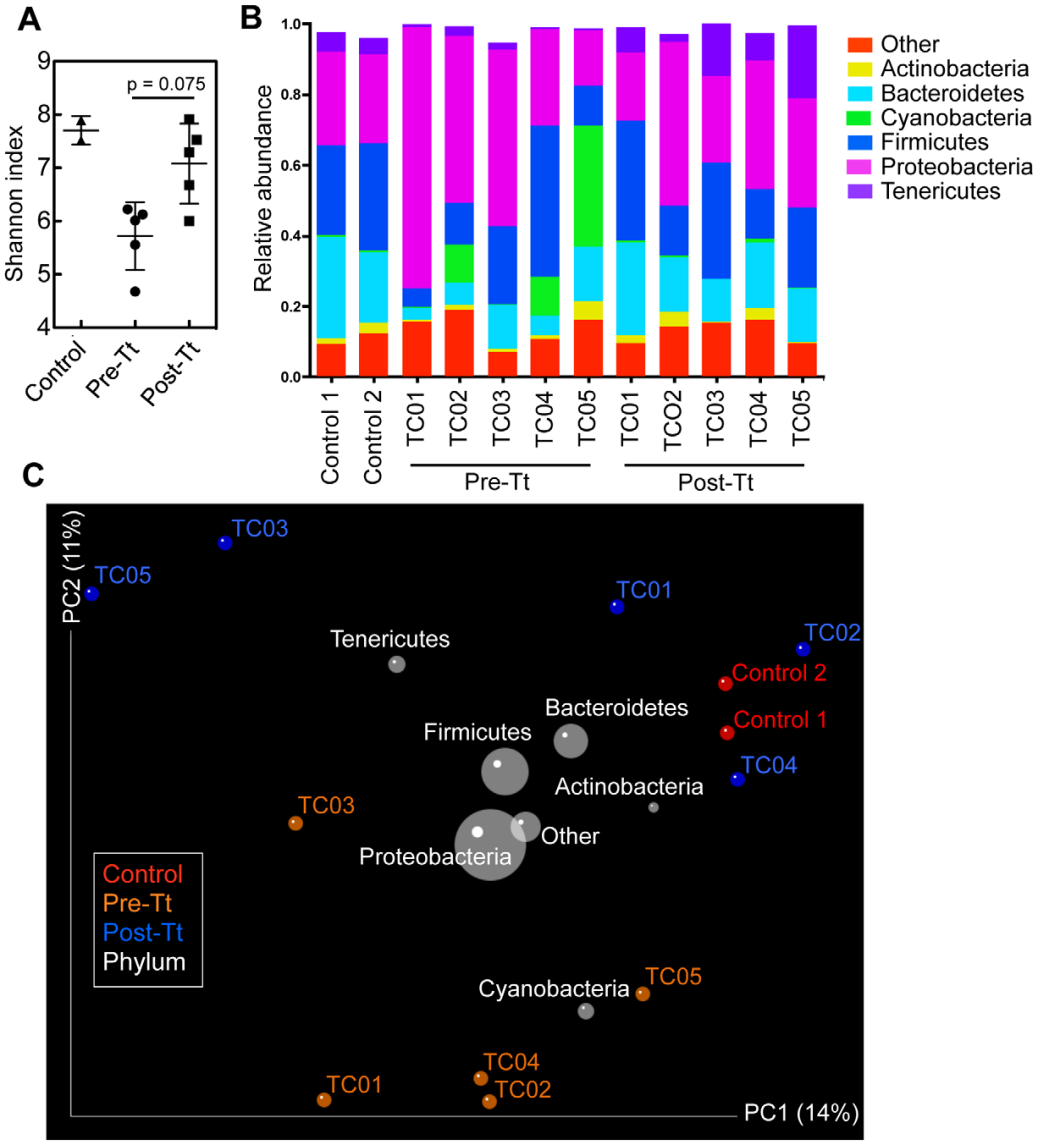
*T. trichiura* treatment influences microbial diversity within samples (α-diversity) and community diversity between samples (β-diversity). (**A**) Shannon diversity of samples from all treatment groups, where each point represents a subject. The average of ten iterations of rarefied subsets of 5422 sequences was used to calculate each Shannon index. The difference between pre- and post-treatment diversity was determined using a Paired Student's t-test (p = 0.075). (**B**) Relative abundance of taxonomic groups for each macaque sample. The seven most abundant phyla over all samples are presented. (**C**) The contribution of different taxonomic groups to the separation of samples based on phylogenetic information. Contributions are represented by the size of the circles (grey) overlaid onto a PCoA of unweighted UniFrac distances of macaques pre-treatment (orange), macaques post-treatment (blue), and controls (red).

In addition to an increased bacterial load, changes to bacterial taxa may be linked with disease pathogenesis. To investigate the composition of microbial communities attached to the intestinal mucosa through culture-independent methods, deep sequencing analysis was performed on the variable region 4 (V4) region of bacterial 16S rRNA [31]. On average, 8439±1373 (SD) sequences were obtained per sample. The microbial diversity within each sample (α-diversity) was compared between biopsies taken from control macaques and from macaques pre-treatment and post-treatment by measuring the Shannon index and phylogenetic diversity (PD) through rarefaction curves (Figure 6A and S5). Shannon diversity was higher in samples post-treatment compared to pre-treatment samples [although not statistically significant (P = 0.0752)] and similar to control samples, suggesting that helminth treatment promoted the restoration of the diversity of mucosal microbial communities.

We then performed a Principal Coordinates Analysis (PCoA) on the unweighted UniFrac distances between samples to analyze differences between microbial communities (β-diversity). We observed clustering of control samples with post-treatment samples and the separation of these samples with pre-treatment samples along the PC2 axis (Figure 6C). Clustering patterns generated with Unweighted Pair Group Method with Arithmetic mean (UPGMA) trees were consistent with the PCoA plot (Figure S5). Interestingly, the non-responder TC05 was found to cluster separately from the other macaques both pre- and post-treatment. By overlaying taxonomic information onto the PCoA plot, we could illustrate that the phyla Bacteroidetes, Firmicutes, and Tenericutes contribute towards the clustering of control and post-treatment samples, whereas Cyanobacteria contributes towards the clustering of the pre-treatment samples (Figure 6C). Indeed, it was striking to find that in three out of the five macaques pre-treatment samples, the bacterial phylum Cyanobacteria was vastly increased in abundance, representing between 10.8–32.9% of total sequences (Figure 6B). Importantly, this taxon is no longer a prominent population post *T. trichiura* treatment. A single operational taxonomic unit (OTU), most closely related to plastids from plant organisms (*Chloroplast: Streptophyta*), constituted 99.2–99.6% of sequences from the *Cyanobacteria* phyla. BLASTN analysis of the sequence against the nr (NCBI) nucleotide database confirmed sequence identity with *Pinus* chloroplast.

**Figure 7:**
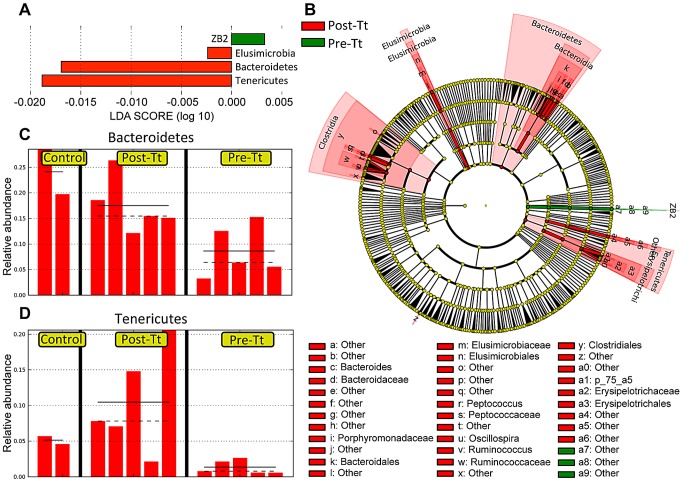
*T. trichiura* treatment recovers several microbial clades reduced in colitic macaques. Utilizing the LEfSe visualization modules and analytical tools, pre-treatment and post-treatment samples were assigned as classes for comparison. (**A**) Histogram of LDA scores computed for features differentially abundant between macaques pre- and post-treatment. (**B**) Cladogram illustrating the taxonomic representation of bacterial taxa with different abundance values in macaques pre- and post-treatment, as determined by LEfSe. Taxa that are more abundant in post-treatment samples are illustrated in red and taxa that are more abundant in pre-treatment samples are illustrated in green. Yellow circles delineate non-significant taxa (**C, D**) Histograms of relative abundances of *Bacteroidetes* (**C**) and of *Tenericutes* (**D**) in control macaques and macaques, pre- and post-treatment.

To further identify bacterial clades with statistically significant differences between control, pre-treatment, and post-treatment samples, we used the LEfSe method [32] for comparison between these groups (Figure 7 and S6). By defining pre-treatment and post-treatment samples as two classes of microbial communities, we found that Tenericutes and Bacteroidetes are more abundant in control and post-treatment samples (consistent with the PCoA plot) whereas the unclassified bacteria taxon ZB2 appears to be slightly enriched in pre-treatment samples (Figure 7A–C). When we compared control with post-treatment samples (Figure 7D and S6), we found that Tenericutes is the only taxon more abundant in post-treatment samples, indicating that it could be induced in response to *T. trichiura* treatment.

## Discussion

**Figure 8:**
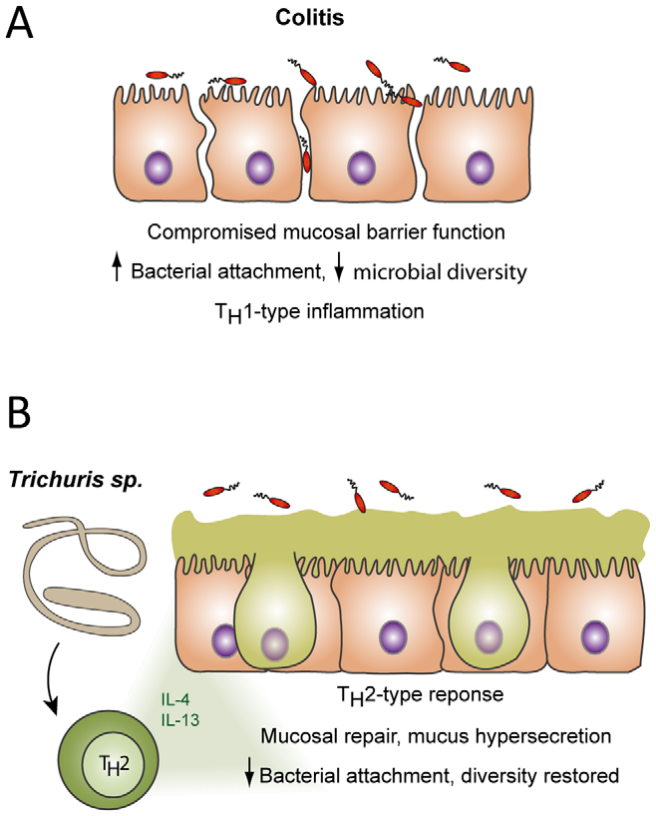
Working model of the immunologic mechanisms underlying the amelioration of colitis in the setting of *Trichuris sp. treatment.* (**A**) Colitis is driven by a T_H_1-type inflammatory response to increased bacterial attachment and dysbiosis at the mucosal epithelium as a result of compromised barrier function. (**B**) *Trichuris sp.* elicits a mucosal T_H_2-type response (including the canonical cytokines IL-4 and IL-13) that promotes mucosal wound healing and mucus production. These functions reduce bacterial attachment and restore microbial homeostasis, removing the inflammatory stimulus.

The data presented here suggest that ICD in juvenile rhesus macaques is an IBD-like disease in which the mucosal barrier is compromised, allowing increased bacterial attachment that contributes to persistent inflammation and dysbiosis of the mucosal microbiota. This study revealed that *T. trichiura* treatment induces a T_H_2-type immune response in the intestinal mucosa of ICD subjects that was associated with symptomatic improvement. This positive clinical response was inversely correlated with cellular markers of mucosal T cell inflammation, such as Ki67^+^ T cells. In contrast to the T_H_2-type response, T_reg_ expansion was noted under conditions of mucosal inflammation and was reduced by *T. trichiura* treatment, suggesting that the presence of T_regs_ in the intestinal tract is more indicative of ongoing intestinal inflammation than of helminth exposure. Based on these results, we speculate that *T. trichiura* promotes mucosal healing in the setting of ICD by activating mucus production and epithelial cell turnover, aspects of T_H_2-type immunity aimed at expelling the parasite [9], [11], [21], thereby reducing the attachment of immunostimulatory bacteria to the colonic epithelium and restoring diversity to the mucosal micobiota (Figure 8). However, additional studies are needed to determine the mechanism of action for *Trichuris*-mediated amelioration of colitis, and functional studies to establish causation would have to be performed in a suitable mouse model to confirm these hypotheses.

One weakness of this study is the absence of a control arm to ensure that the macaques with ICD did not undergo spontaneous remission. Future studies to confirm the findings presented here should have a sham-treated control arm, which would also enable a blinded assessment of stool frequency. The association of clinical improvement with weight gains, which is an objective measure, supports the observation that *T. trichiura* treatment did lead to improvement in symptoms. Additionally, our transcriptional profiling experiments clearly show that clinical improvement is associated with dramatic changes in mucosal gene expression patterns. The number of biopsies that could be collected from these juvenile macaques also limited our study. Histopathological observations that could be correlated with gene expression patterns would have provided additional insights into the relationship between cellular infiltrates and mucosal responses. While ICD shares some similar features with UC, inflammation is typically microscopic and cannot be determined objectively by visual inspection during endoscopy.

The mucosal gene expression patterns (e.g., REG1, REG3, NOS2, TFF1, SAA1, and SAA3) that distinguish ICD and healthy macaques are strikingly similar to signatures observed in microarray studies of UC patients [12], [26], [33], [34] as well as in studies of germ free mice repopulated with various bacterial taxa [35], [36], suggesting that a response to gut bacteria may be the predominant driving force for this inflammatory signature. Importantly, differences in gene expression patterns were apparent between the four clinical responders and the single non-responder in this study. Of note, the non-responder showed lower expression of immunoglobulin-related genes (Figure S4), suggesting that the etiology of disease in this animal may have stemmed from a defect in the mucosal B cell compartment, as has been shown previously to precipitate colitis through the absence of regulatory B cells [37]. Recently, MyD88 signaling in B cells was found to prevent the lethal dissemination of intestinal bacteria after dextran sulfate sodium (DSS) treatment in mice [38].

The IL-23, IL-17 and IL-22 network plays a critical role in intestinal homeostasis and IBD pathogenesis [39]. We previously reported that IL-22-producing CD4^+^ cells were a prominent feature of *T. trichiura* infection in a UC patient. Unfortunately, our efforts to stain for IL-17- and IL-22-producing CD4^+^ cells from the macaque pinch biopsies were unsuccessful (data not shown). When we investigated the expression levels of the cytokines in these pathways pre- and post-*T. trichiura* treatment, we found no significant trends (Figure S7). Since the role of these cytokines in ICD of macaques is completely unknown, this may represent an important difference with human IBD, but it is difficult to draw any conclusions from these results at the moment.

The effects of *T. trichiura* treatment on improving symptoms of colitis occurred despite the absence of a chronic and active infection. Ova were never detected in fecal samples from any of the macaques, indicating the lack of a patent infection, and adult worms were not seen during the post-treatment colonoscopy. The lack of patency is most likely due to a species barrier, since the *T. trichiura* ova used for inoculation were from a human subject [12]. Notably, this parallels the use of *T. suis* ova (TSO) as a therapeutic intervention in human subjects with autoimmune diseases, which is dependent on a species barrier to eliminate the parasites after dosing. After hatching, *Trichuris* larvae molt several times in the intestines before maturing into adult worms. Thus, the larval stages could have elicited a mucosal response leading to a positive clinical outcome. The absence of egg production does not preclude the presence of adult forms or mature larval forms, although none were observed during the endoscopies. Despite the absence of adult forms in humans, TSO clearly induces a strong T_H_2 response in treated subjects, as indicated by increased serum IL-4, eosinophilia, and IgE antibodies [40], [41]. Although we did not detect more IL-4-producing CD4^+^ T cells by intracellular cytokine staining in post-treatment PBMCs, this is not indicative that a systemic Th2 response was absent in the macaques. In our previous study [12], responses in PBMCs could only be detected after antigen-specific expansion of CD4^+^ cells with *T. trichiura* antigen. We propose that the treatment of colitis (but not necessarily other autoimmune diseases) with *Trichuris sp.* may be more dependent on a T_H_2 response than immunoregulation. Since we did not detect ova in the feces of the treated macaques, it is also possible that the clinical improvements were spontaneous and not related to *T. trichiura* treatment. However, we find this unlikely since the T_H_2 response in the intestinal mucosa at a cellular and molecular level is remarkably consistent among all of the treated macaques.

It is important to note the changes in absolute bacterial quantities as well as the composition of mucosal microbiota following helminth exposure. With both measures, post-treatment samples were much more similar to samples from healthy controls. Perhaps the most consistent observation made in studies on the microbiota of IBD patients is the overall reduction in microbial diversity [23]. There is also reduced diversity of the intestinal microbiota in rhesus macaques with colitis, as noted previously [25] as well as in this study, supporting the use of rhesus macaques with ICD as a preclinical model for IBD.

We noted a striking expansion of Cyanobacteria in three ICD subjects, which disappeared post-treatment. Cyanobacteria have been previously noted to reside in the intestinal tract of mice and humans [42] as well as that of macaques [25]. However, there have not been any previous reports, including studies on the mucosal microbiota from IBD patients [26], where this taxon has represented an abundance of up to 32.9% of 16S sequences. This expansion may reflect a unique environment on the intestinal mucosa of juvenile rhesus monkeys with chronic diarrhea. Notably, a recent publication (including data from the Human Microbiome Project) indicated that, in healthy human beings, the taxon Cyanobacteria∶Chloroplast∶Streptophyta was enriched in non-mucosal sites relative to mucosal body sites and also enriched under conditions with high oxygen availability [32]. Like *Salmonella typhimurium* [43], perhaps the activation of neutrophils to produce reactive oxygen species favors the expansion of a Cyanobacterium more adapted to an aerobic environment or to alterations in redox conditions. It is also quite possible that these sequences are merely a representation of plant organisms in poorly digested food, which could be more abundantly attached to the mucosa of monkeys suffering from diarrhea.

Since Bacteroidetes is the most predominant phylum in the normal gut, the reduction in proportional abundance in ICD subjects may reflect an expansion of non-Bacteroidetes phyla amongst the mucosal microbiota during ICD. Indeed, quantification of absolute Bacteroidetes abundance by RT-PCR clearly shows a reduction post-treatment. These results reflect a typical problem for this type of 16S compositional data of microbial communities. What is more interesting is the unexpected expansion of Tenericutes after helminth exposure, even relative to control macaques, indicating that bacteria of this phylum could be preferentially expanded in a T_H_2-type mucosal environment. Little is known about the role of Tenericutes in the intestinal tract. In one previous study with mice, dextran sodium sulfate (DSS)-driven colitis was shown to decrease the abundance of this phylum [44]. Further studies on the relationship between intestinal helminths and Tenericutes are warranted.

ICD-afflicted juvenile rhesus macaques may represent a useful preclinical model in which to further study the pathology, diagnosis, and treatment of UC in humans under certain circumstances. Most notably, the transcriptional signature of the inflamed mucosa in these macaques closely resembles that of UC patients. Given the prevalence of ICD at primate research centers, this could be an important resource to develop new therapeutics for IBD in humans, especially when addressing the need of a non-rodent model during preclinical testing. However, the costs and ethical issues of using non-human primates may limit the usefulness of this animal model for IBD research towards very specific circumstances when an outbred non-rodent model is required. While very similar, there are also distinct differences between the macaque immune system and the human immune system [45], [46] that may limit the use of ICD afflicted rhesus macaques as a model for IBD research.

Our findings describe the immune mechanisms that may mediate the therapeutic effect of *Trichuris sp.* in the setting of colitis and highlight the role of T_H_2-type immunity in promoting mucosal repair. Instead of acting through an immunoregulatory mechanism, *Trichuris sp.* may trigger a T_H_2-driven expulsion mechanism related to increased mucus production and turnover of epithelial cells, thereby reducing the quantity of attached bacteria among the mucosal microbiota. As part of this process, the dysbiosis observed in the mucosal environment during colitis could also revert back to a normal equilibrium.

## Materials and Methods

### Ethics statement

This study was carried out in strict accordance with the recommendations in the Guide for the Care and Use of Laboratory Animals of the National Institutes of Health. The use and care of all animals followed policies and guidelines established by the University of California, Davis Institutional Animal Care and Use Committee (IACUC) and CNPRC (Animal Welfare Assurance #A3433-01). The protocol for this trial was approved by the University of California, Davis IACUC. Veterinary care was provided to all animals in order to minimize pain and distress. The California National Primate Research Center (CNPRC), which houses over 5,000 nonhuman primates, is a part of the National Primate Research Centers Program and is accredited by the Association for the Assessment and Accreditation of Laboratory Animal Care, International (AAALAC). All animal facilities are maintained in compliance with United States Department of Agriculture specifications. Rhesus macaques are maintained in small or large social groups.

### Subject recruitment and clinical monitoring

All animals were housed indoors at the CNPRC. ICD cases were identified by recurrent episodes of diarrhea (during 45 or more days in a 90 day period) without evidence for (or a history of) known causes of infectious colitis, as demonstrated by three negative cultures for bacterial pathogens and negative stool examination and immunofluorescence assays for protozoan and helminthic parasites. The diarrhea was refractory to antibiotic and antiparasitic treatment. Animals were weighed every 8–14 days and their stool evaluated according to a standardized four-point scale for fecal consistency (Figure 1).

### Collection of colonic biopsies and peripheral blood

Animals were fasted for 36 hours prior to colonoscopy and 30 mL/kg of polyethylene glycol-electrolyte solution (PEG-ES; GoLYTELY brand) was provided twice the day before the procedure by mixing 67 gm with 1 liter of citrus-flavored water (Tang, Kraft Foods, Northfield, IL) and making the solution available for the animals to drink by a hanging bottle in the cage. Blood was collected by venipuncture into citrate tubes prior to colonoscopy. Five pinch biopsies were collected during colonoscopy from the proximal ascending colon. Three biopsies were collected into culture media for *ex vivo* analysis and two biopsies were collected into RNAlater (Qiagen) for nucleic acid extraction.

### Flow cytometric analysis of biopsies and peripheral blood

Colon biopsy specimens were treated with 0.25 mg/ml collagenase type II (Sigma-Aldrich) for 30 minutes with constant shaking at room temperature. Digested tissue was dispersed over a 70-micron nylon mesh filter. Cell suspensions were washed twice with RPMI containing 15% fetal calf serum. Whole blood was collected into ACD-containing tubes (BD Biosciences) and PBMC were isolated by density centrifugation. Biopsy cells and PBMC (1×10^6^) were resuspended in 200 µl of complete R-10 [RPMI 1640 medium (Invitrogen) supplemented with 10% fetal calf serum (Hyclone), 50 U/ml penicillin, 50 µg/ml streptomycin, and 2 mM L-glutamine], and stimulated with phorbol myristate acetate (10 ng/ml) and ionomycin (1 µg/ml) in the presence of brefeldin A (GolgiPlug, BD Pharmingen) for five hours at 37°C. For biopsy cells, amphotericin B (Gibco) was also added to the culture media. Cell surface staining and intracellular cytokine staining were performed with Fix/Perm and PermWash solutions from BD and eBioscience, according to the manufacturer's instructions.

### Microarray analysis of biopsy samples

Biopsies were collected into RNAlater (Qiagen) and homogenized in TRIzol (Invitrogen). RNA was collected in the aqueous extraction phase and column purified using an RNeasy kit (Qiagen). Sample preparation, labeling, and array hybridizations were performed according to standard protocols from the UCSF Shared Microarray Core Facilities and Agilent Technologies (http://www.arrays.ucsf.edu and http://www.agilent.com). Total RNA quality was assessed using a Pico Chip on an Agilent 2100 Bioanalyzer (Agilent Technologies, Palo Alto, CA). RNA was amplified and labeled with Cy3-CTP using the Agilent low RNA input fluorescent linear amplification kits following the manufacturer's protocol (Agilent). Labeled cRNA was assessed using the Nandrop ND-100 (Nanodrop Technologies, Inc., Wilmington DE) and equal amounts of Cy3 labeled target were hybridized to Agilent Rhesus Macaque (V2) whole genome 4x44K Ink-jet arrays (Agilent). Hybridizations were performed for 14 hours, according to the manufacturer's protocol (Agilent). Arrays were scanned using the Agilent microarray scanner (Agilent) and raw signal intensities were extracted with Feature Extraction v10.1 software (Agilent). Each data set was normalized using the *quantile* normalization method [47] with no background subtraction. A one-way ANOVA linear model was fit to the comparison to estimate the mean M values and calculated B statistic, false discovery rate and p-value for each gene for the comparison of interest. All procedures were carried out using functions in the R package. For DAVID [48] and PANTHER [49] pathway analyses, EnsEMBL and Genbank accession numbers associated with significantly different *M. mulatta* genes were converted to official gene symbols, along with a background list consisting of all genes used as input to the analyses. Functional annotation charts were generated using the default parameters. Reported p-values are Bonferroni-corrected. For these analyses, the human pathways and ontologies were used as they are more fully developed than those available for macaques.

### RT-PCR analysis of RNA and DNA from colon biopsies

Tissue samples were homogenized in TRIzol (Invitrogen). RNA was collected in the aqueous extraction phase, and DNA was harvested from the interphase and phenol-chloroform organic phase. RNA was column purified using an RNeasy kit (Qiagen). cDNA was generated using an Omniscript Reverse Transcription kit (Qiagen) with oligo-dT primers in the presence of RNasin Plus RNase inhibitor (Promega). DNA was collected by ethanol precipitation and washed according to the manufacturer's instructions. PCR reactions were carried out with Taqman primer/probe sets (Applied Biosystems) in a StepOne Plus machine (Applied Biosystems). The abundances of major intestinal bacterial groups were measured by qPCR of extracted DNA, using the MyIQ single-color real-time PCR detection system (Bio-Rad, Hercules, CA) and group-specific 16S rRNA gene primers that have been previously published and verified [50]. Samples were normalized to controls and eubacteria.

### Soluble CD14 analysis

Plasma sCD14 levels were measured using the Quantikine ELISA kit (R&D Systems; Minneapolis, Minnesota, USA), as described in the manufacturer's protocol. Plasma samples were diluted 1/500 and run in duplicate.

### 16S rRNA analyses

DNA samples isolated from pinch biopsies were PCR amplified for sequencing through a validated protocol [31]. The V4 region of the 16S rRNA gene was amplified with region specific barcoded primers [51] and sequenced on a MiSeq sequencer [31] along with other barcoded samples. Reads shorter than 140 bp were discarded. The QIIME suite of analysis tools was used to filter and analyze the sequence data [52]. Sequences were assigned to OTUs with a threshold of 97% pair-wise identity and then classified taxonomically using the Ribosomal Database Project (RDP) classifier. A representative sequence for each OTU was aligned using PyNAST and used to build a phylogenetic tree for α-diversity and β-diversity measurements. Alpha rarefaction was performed using the Shannon index and phylogenetic diversity. Beta diversity was calculated in QIIME, using default metrics of weighted and unweighted UniFrac distances between samples, and visualized using Principal Coordinate Analysis (PCoA) plots and Unweighted Pair Group Method with Arithmetic Mean (UPGMA) trees. Relative abundance of microbial phyla was determined in QIIME by grouping OTUs by macaque sample. To identify significant differences in bacterial taxa between groups, we utilized the linear discriminant analysis (LDA) effect size (LEfSe) algorithm [32] through the Galaxy Framework [53], [54] online. LEfSe uses the Kruskal-Wallis (KW) sum-rank test to detect features with significantly different abundances between classes, and performs LDA to estimate the effect size of each differentially abundant feature. So that we could recover all features detected by the KW test, we did not set a threshold for LDA in the analysis.

## Supporting Information

Figure S1 **Spontaneous remission does not occur in untreated juvenile macaques with chronic diarrhea.** Fecal consistency scores (FCS) were based on a standardized 4-point scale; 1 = Well-formed, normal; 1.5 = Normal to semi-solid; 2 = Semi-solid to normal; 2.5 = Semi-solid; 3 = Semi-solid to liquid; 3.5 = Liquid to semi-solid; 4 = Liquid. Semi solid stool is defined as “porridge-like” or able to be picked up with a fork. Data is shown as the average and SEM of N = 7 subjects.
(TIFF)

Figure S2 **Hierarchical clustering of genes differentially expressed in colon biopsies between colitis subjects [before (Pre) and after (Post)** ***T. trichiura*** **treatment] and healthy controls (Cont) as shown in** **Figure 1** **, with gene symbols listed.**
(TIF)

Figure S3 **Hierarchical clustering of genes differentially expressed in colon biopsies following** ***T. trichiura*** **treatment in clinical responders (R) and subject TC05 (NR) as shown in** **Figure 1** **, with gene symbols listed.**
(TIF)

Figure S4 **Hierarchical clustering of genes differentially expressed between clinical responders (R) and subject TC05 (NR) pre-treatment, with gene symbols listed.** Probe ID's are shown where gene symbols are not available. Additional annotation indicates probes that represent Immunoglobulin genes that are down regulated in expression in subject TC05 (NR).
(TIF)

Figure S5 ***T. trichiura*** **treatment influences microbial diversity within samples (α-diversity) and community diversity between samples (β-diversity).** (**A, B**) Rarefaction plots of Shannon diversity (**A**) and phylogenetic diversity (**B**). The average of ten iterations of rarefied subsets was used to calculate each metric. Metrics are averaged across macaques within each treatment and error bars represent the standard deviation. (**C**) Phylogenetic diversity of each macaque sample, calculated by the average of ten iterations of rarefied subsets of 5422 sequences. The difference between pre- and post-treatment diversity was determined using a Paired Student's t-test (p = 0.178). (**D, E**) UPGMA trees of unweighted UniFrac (**D**) and weighted UniFrac (**E**). Branch colors: macaques pre-treatment (orange), macaques post-treatment (blue), and controls (red).
(TIF)

Figure S6 ***T. trichiura*** **treatment increases the relative abundance of bacteria from the phyla** ***Tenericutes*** **in treated macaques compared to control healthy macaques.** Utilizing LEfSe, post-treatment samples and control samples were assigned as classes for comparison. The cladogram illustrates the taxonomic representation of bacterial taxa with different abundance values in control macaques and post-treatment macaaques. Taxa that are more abundant in control samples are illustrated in red and taxa that are more abundant in post-treatment samples are illustrated in green. Yellow circles delineate non-significant taxa.
(TIF)

Figure S7 **Expression of T_H_17/22 cytokine genes from pinch biopsies pre-treatment (Pre-Tt) and post-treatment (Post-Tt) showed no significant trends.** Expression levels of genes in pre-treatment (Pre-Tt) and post-treatment (Post-Tt) colitis subjects were extracted from the microarray dataset. Expression levels of IL-17 were confirmed by RT-PCR (data not shown). Samples from subject TC05 are shown in red.
(TIF)

Table S1 **List of genes differentially expressed in colon biopsies between colitis subjects and healthy controls.** Genes most highly expressed in colitis subjects are listed at the top. FDR, false discovery rate.
(PDF)

Table S2 **List of genes differentially expressed in colon biopsies from colitis subjects before and after** ***T. trichiura*** **treatment.** Genes most highly expressed following treatment are listed at the top. FDR, false discovery rate.
(PDF)

Table S3 **List of genes differentially expressed in colon biopsies between clinical responders (R) and subject TC05 (NR) pre-treatment with** ***T. trichiura*** **.** Genes most highly expressed in the non-responding subject TC05 are listed at the top. FDR, false discovery rate.
(PDF)
