# Supplementary figures and images for: Therapeutic Helminth Infection of Macaques with Idiopathic Chronic Diarrhea Alters the Inflammatory Signature and Mucosal Microbiota of the Colon
Source: PLoS Pathog. 2012 Nov 15;8(11):e1003000. doi: 10.1371/journal.ppat.1003000 (PMC3499566; doi:10.1371/journal.ppat.1003000)

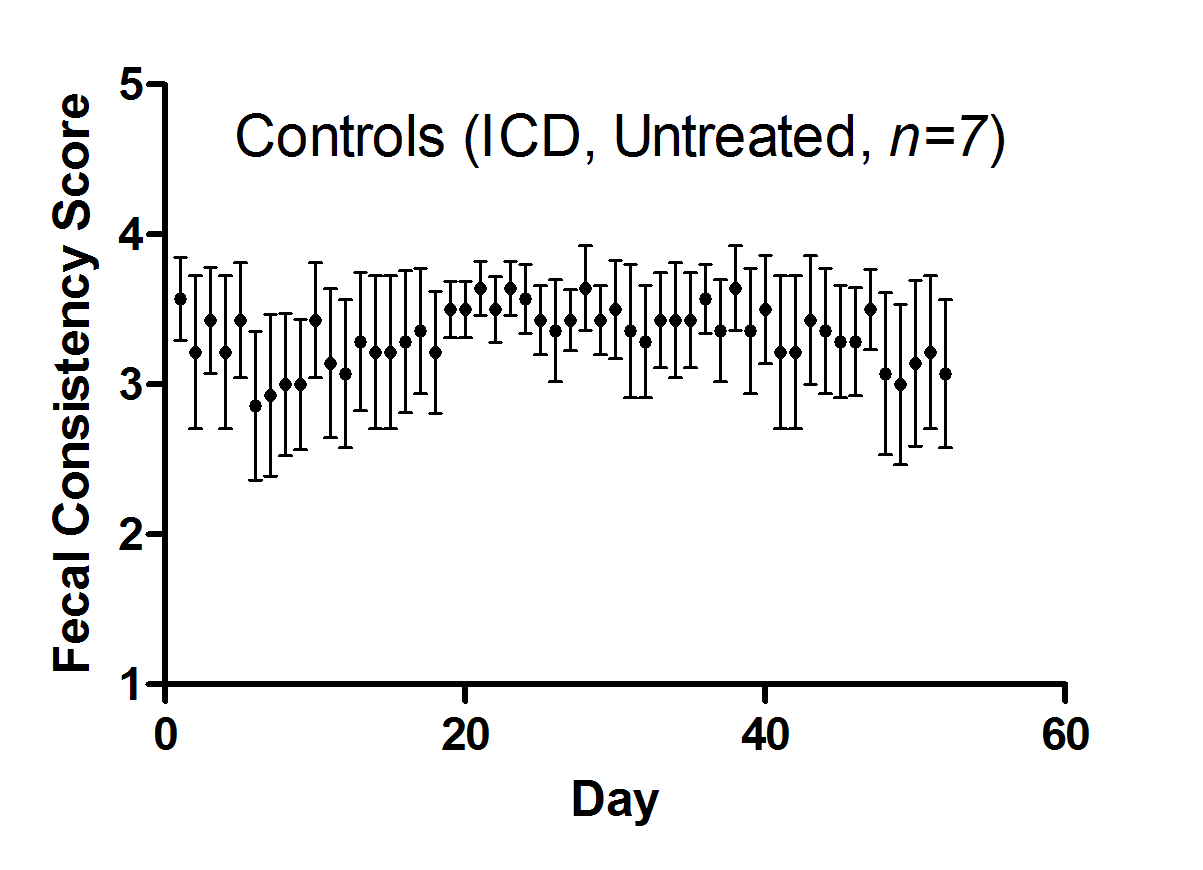

Supplement: Figure S1 — Spontaneous remission does not occur in untreated juvenile macaques with chronic diarrhea. Fecal consistency scores (FCS) were based on a standardized 4-point scale; 1 = Well-formed, normal; 1.5 = Normal to semi-solid; 2 = Semi-solid to normal; 2.5 = Semi-solid; 3 = Semi-solid to liquid; 3.5 = Liquid to semi-solid; 4 = Liquid. Semi solid stool is defined as “porridge-like” or able to be picked up with a fork. Data is shown as the average and SEM of N = 7 subjects. (TIFF) [file ppat.1003000.s001.tif]

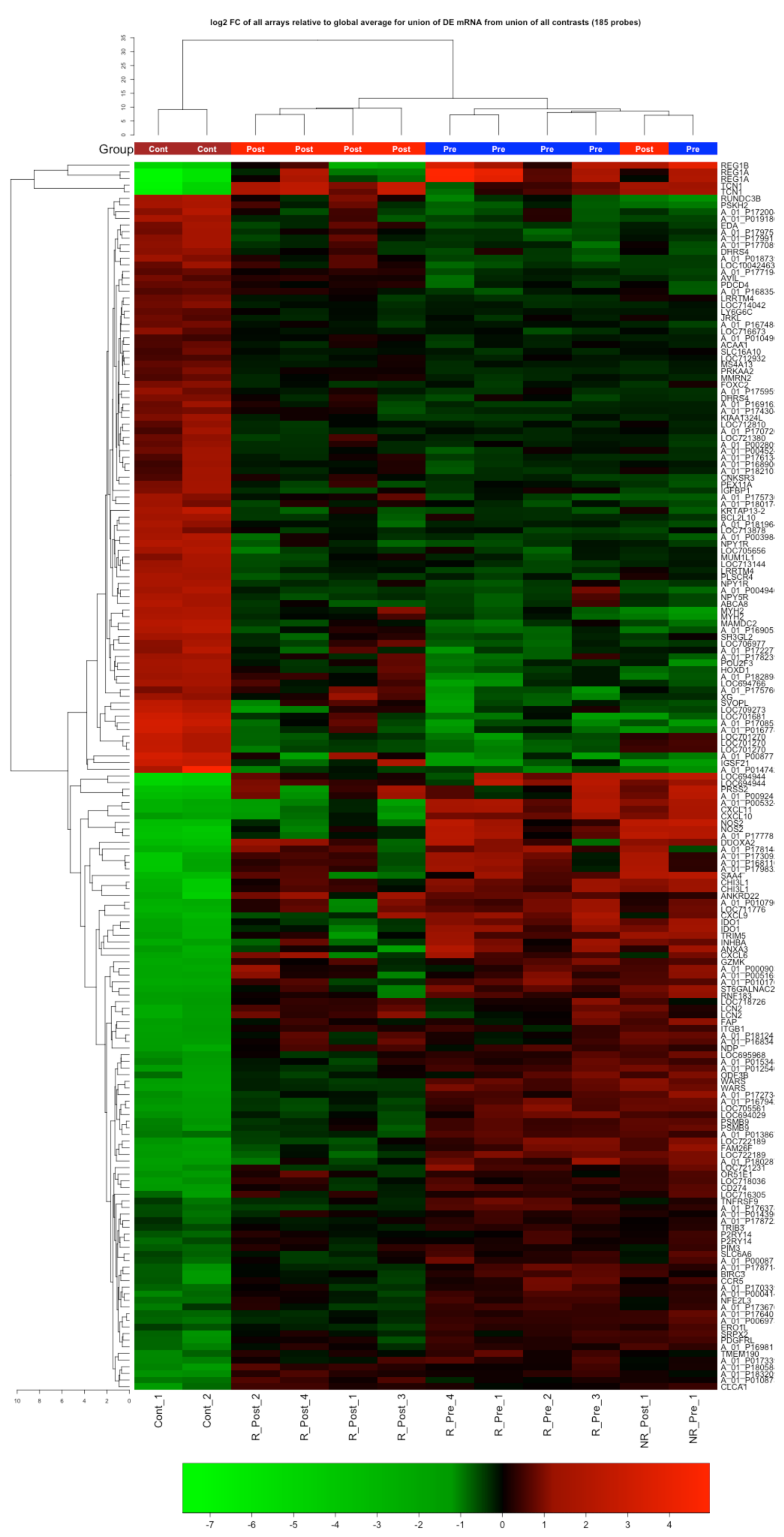

Supplement: Figure S2 — Hierarchical clustering of genes differentially expressed in colon biopsies between colitis subjects [before (Pre) and after (Post) T. trichiura treatment] and healthy controls (Cont) as shown in Figure 1 , with gene symbols listed. (TIF) [file ppat.1003000.s002.tif]

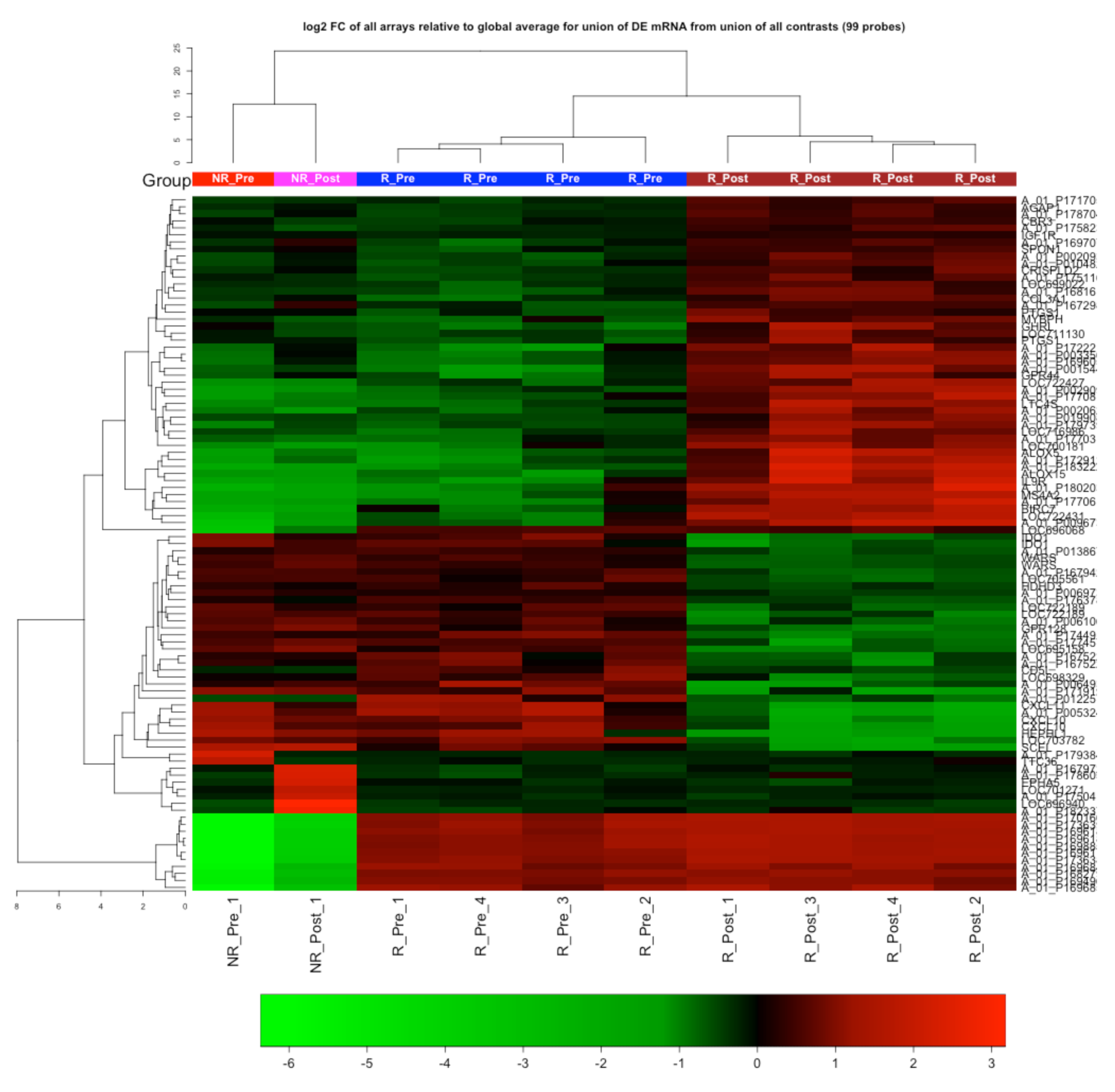

Supplement: Figure S3 — Hierarchical clustering of genes differentially expressed in colon biopsies following T. trichiura treatment in clinical responders (R) and subject TC05 (NR) as shown in Figure 1 , with gene symbols listed. (TIF) [file ppat.1003000.s003.tif]

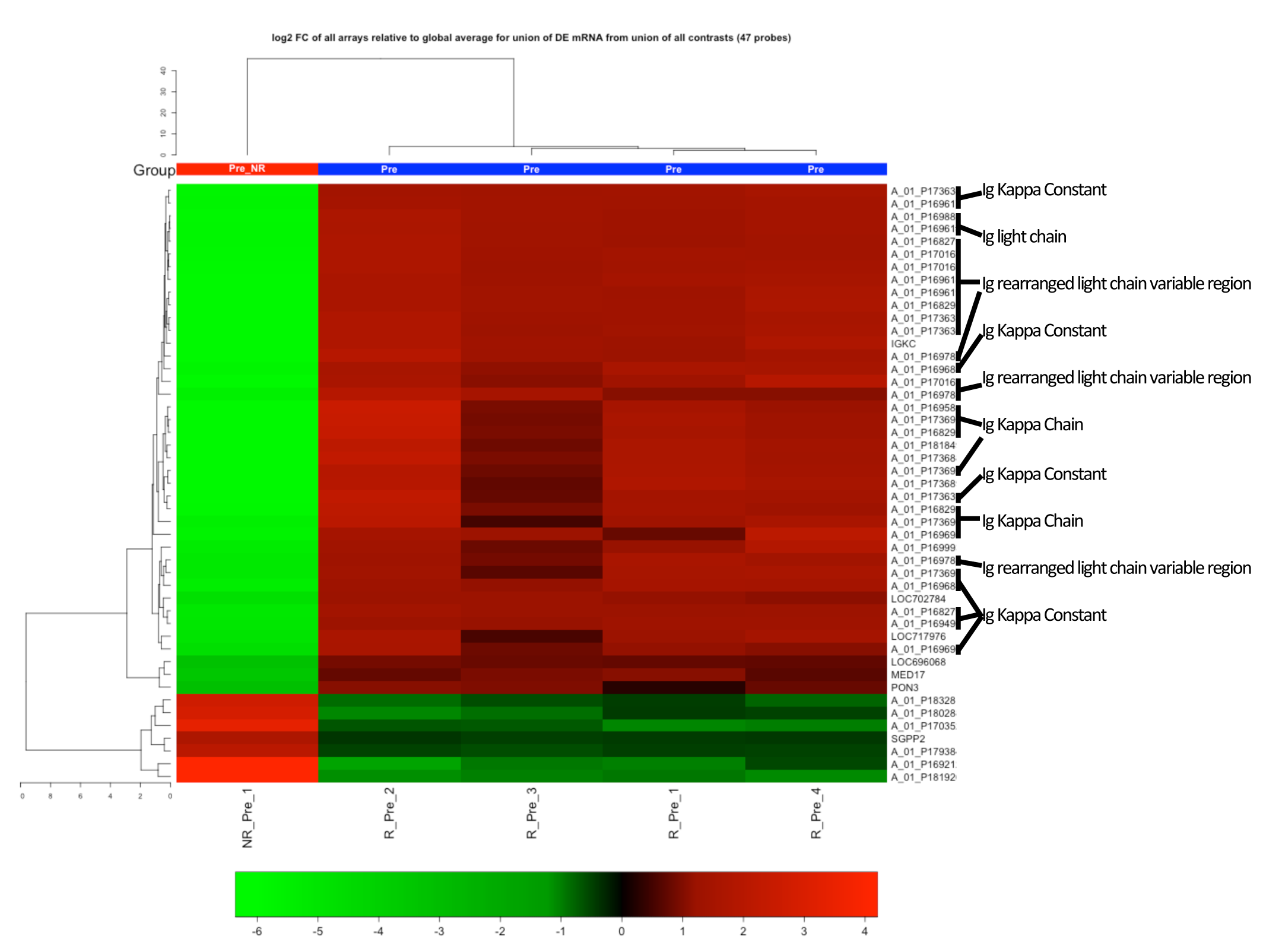

Supplement: Figure S4 — Hierarchical clustering of genes differentially expressed between clinical responders (R) and subject TC05 (NR) pre-treatment, with gene symbols listed. Probe ID's are shown where gene symbols are not available. Additional annotation indicates probes that represent Immunoglobulin genes that are down regulated in expression in subject TC05 (NR). (TIF) [file ppat.1003000.s004.tif]

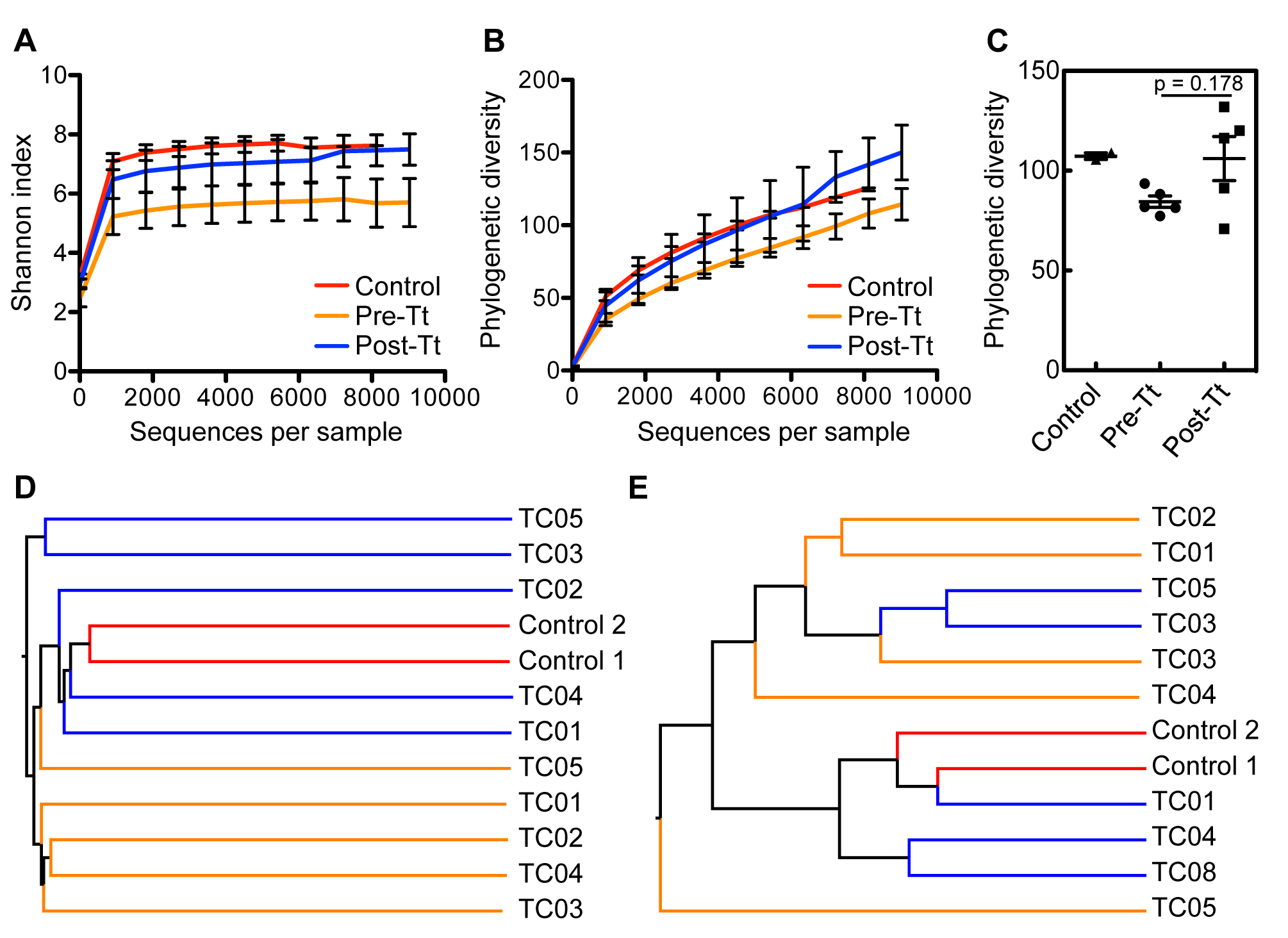

Supplement: Figure S5 — T. trichiura treatment influences microbial diversity within samples (α-diversity) and community diversity between samples (β-diversity). (A, B) Rarefaction plots of Shannon diversity (A) and phylogenetic diversity (B). The average of ten iterations of rarefied subsets was used to calculate each metric. Metrics are averaged across macaques within each treatment and error bars represent the standard deviation. (C) Phylogenetic diversity of each macaque sample, calculated by the average of ten iterations of rarefied subsets of 5422 sequences. The difference between pre- and post-treatment diversity was determined using a Paired Student's t-test (p = 0.178). (D, E) UPGMA trees of unweighted UniFrac (D) and weighted UniFrac (E). Branch colors: macaques pre-treatment (orange), macaques post-treatment (blue), and controls (red). (TIF) [file ppat.1003000.s005.tif]

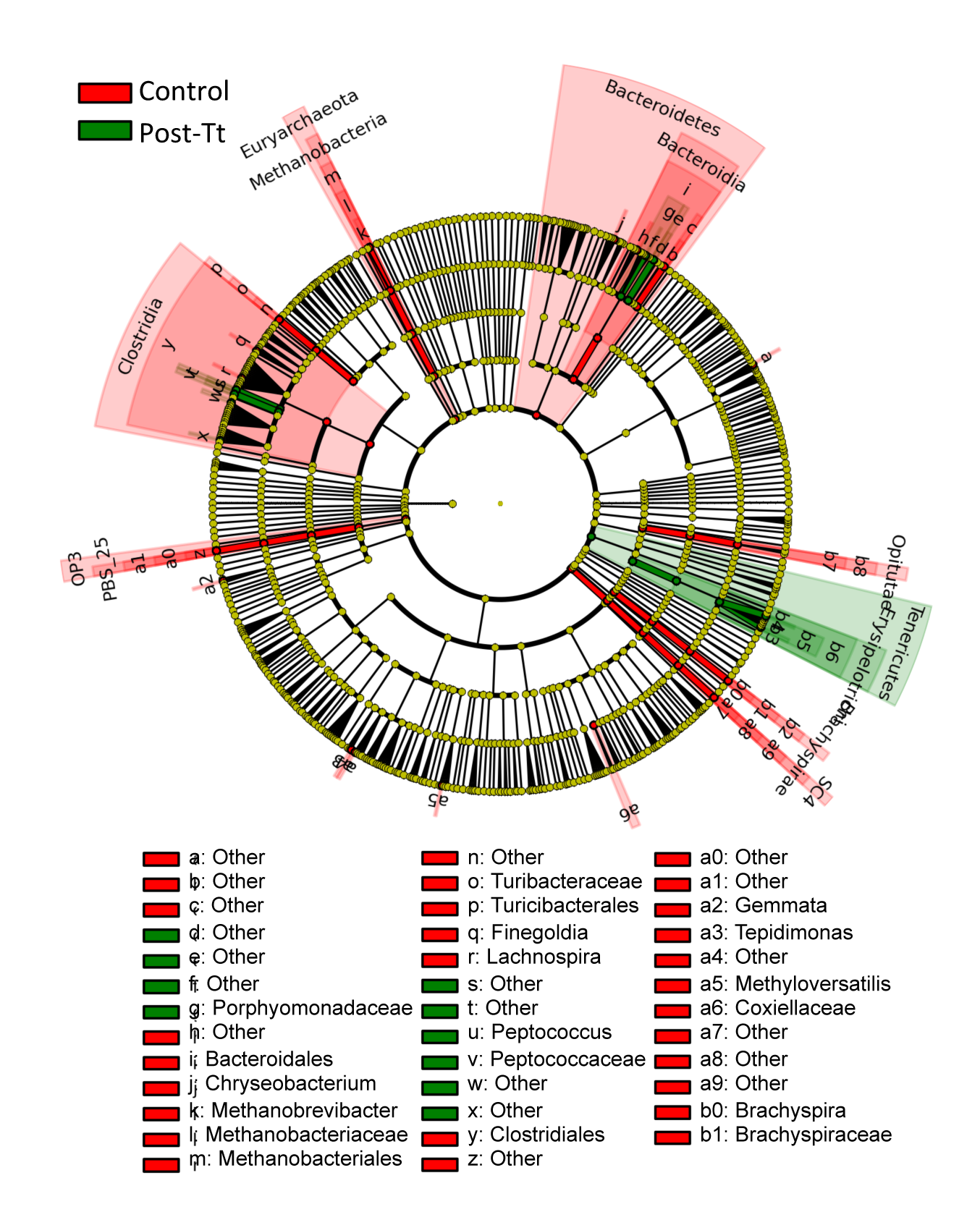

Supplement: Figure S6 — T. trichiura treatment increases the relative abundance of bacteria from the phyla Tenericutes in treated macaques compared to control healthy macaques. Utilizing LEfSe, post-treatment samples and control samples were assigned as classes for comparison. The cladogram illustrates the taxonomic representation of bacterial taxa with different abundance values in control macaques and post-treatment macaaques. Taxa that are more abundant in control samples are illustrated in red and taxa that are more abundant in post-treatment samples are illustrated in green. Yellow circles delineate non-significant taxa. (TIF) [file ppat.1003000.s006.tif]

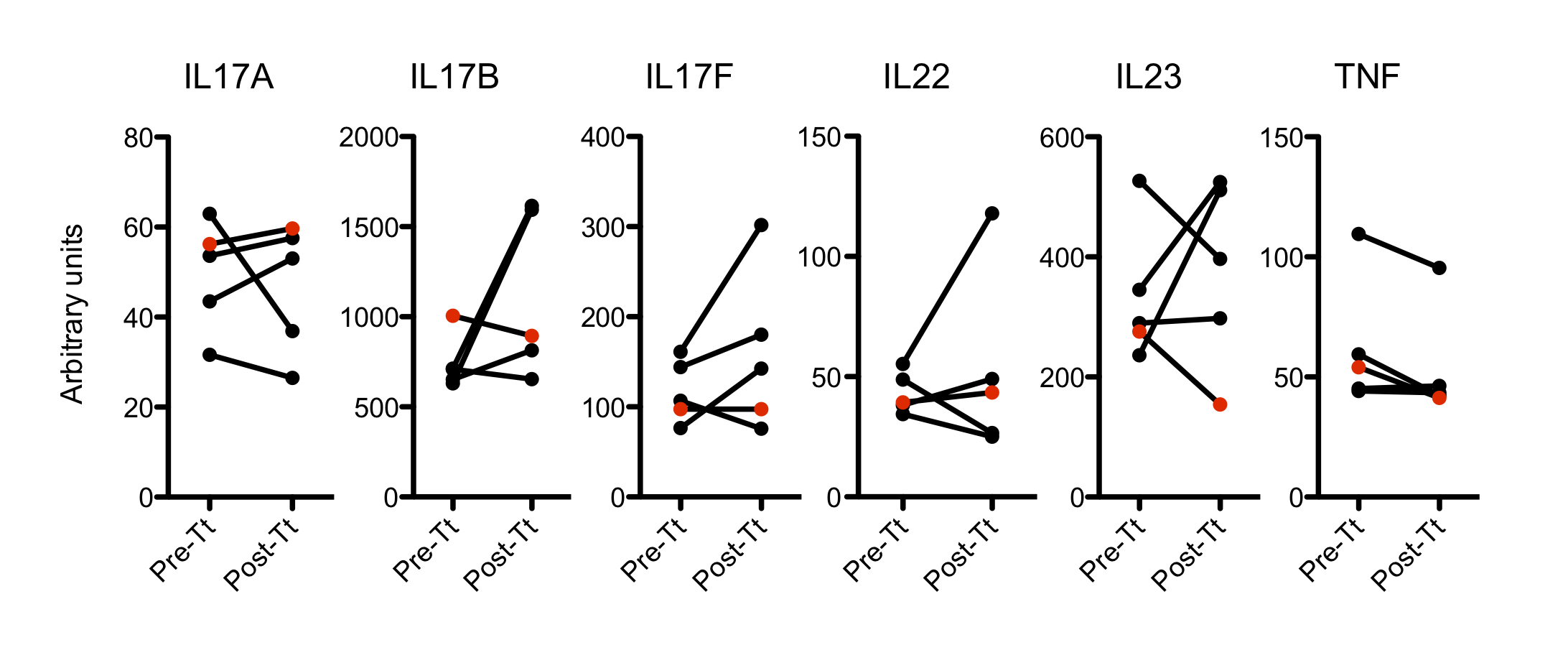

Supplement: Figure S7 — Expression of TH17/22 cytokine genes from pinch biopsies pre-treatment (Pre-Tt) and post-treatment (Post-Tt) showed no significant trends. Expression levels of genes in pre-treatment (Pre-Tt) and post-treatment (Post-Tt) colitis subjects were extracted from the microarray dataset. Expression levels of IL-17 were confirmed by RT-PCR (data not shown). Samples from subject TC05 are shown in red. (TIF) [file ppat.1003000.s007.tif]
