# Supplementary material for: Therapeutic Helminth Infection of Macaques with Idiopathic Chronic Diarrhea Alters the Inflammatory Signature and Mucosal Microbiota of the Colon
Source: PLoS Pathog. 2012 Nov 15;8(11):e1003000. doi: 10.1371/journal.ppat.1003000 (PMC3499566; doi:10.1371/journal.ppat.1003000)
Supplement: Table S1 — List of genes differentially expressed in colon biopsies between colitis subjects and healthy controls. Genes most highly expressed in colitis subjects are listed at the top. FDR, false discovery rate. (PDF) [file ppat.1003000.s008.pdf]

Table S1

Description	Fold	FDR
ILLUMIGEN_MCQ_46808 Katze_MMJJ Macaca mulatta cDNA clone IBIUW:18836 5' similar to Bases 23 to 794 highly similar to human REG1A (Hs.49407), mRNA sequence [CO581740]	500.00	0.005
Macaca mulatta regenerating islet-derived 1 beta (REG1B), mRNA [NM_001194568]	125.00	0.027
transcobalamin I (vitamin B12 binding protein, R binder family) [Source:HGNC Symbol;Acc:11652] [ENSMMUT00000018989]	83.33	0.002
transcobalamin I (vitamin B12 binding protein, R binder family) [Source:HGNC Symbol;Acc:11652] [ENSMMUT00000018989]	76.92	0.003
PREDICTED: Macaca mulatta serum amyloid A protein-like, transcript variant 1 (LOC694944), mRNA [XM_001086477]	71.43	0.002
Amyloid protein A (Amyloid fibril protein AA) [Source:UniProtKB/Swiss-Prot;Acc:P02738] [ENSMMUT00000015626]	71.43	0.006
PREDICTED: Macaca mulatta nitric oxide synthase 2, inducible, transcript variant 4 (NOS2), mRNA [XM_001106245]	38.46	0.010
PREDICTED: Macaca mulatta nitric oxide synthase 2, inducible, transcript variant 4 (NOS2), mRNA [XM_001106245]	27.03	0.047
Macaca mulatta protease, serine, 2 (trypsin 2) (PRSS2), mRNA [NM_001047120]	22.73	0.041
chitinase 3-like 1 (cartilage glycoprotein-39) [Source:HGNC Symbol;Acc:1932] [ENSMMUT00000012174]	21.74	0.002
chitinase 3-like 1 (cartilage glycoprotein-39) [Source:HGNC Symbol;Acc:1932] [ENSMMUT00000012174]	20.83	0.002
Macaca mulatta dual oxidase maturation factor 2 (DUOXA2), mRNA [NM_001194505]	18.18	0.027
serum amyloid A4, constitutive [Source:HGNC Symbol;Acc:10516] [ENSMMUT00000015624]	17.54	0.046
protease, serine, 2 [Source:RefSeq peptide;Acc:NP_001040585] [ENSMMUT00000041633]	17.54	0.041
small inducible cytokine B11 [Source:RefSeq peptide;Acc:NP_001028122] [ENSMMUT00000029393]	16.39	0.021
ankyrin repeat domain 22 [Source:HGNC Symbol;Acc:28321] [ENSMMUT00000000694]	16.13	0.011
Macaca mulatta chemokine (C-X-C motif) ligand 11 (CXCL11), mRNA [NM_001032950]	15.87	0.010
Macaca mulatta chemokine (C-X-C motif) ligand 9 (CXCL9), mRNA [NM_001032936]	14.93	0.006
Macaca mulatta unknown Fragment [Source:UniProtKB/TrEMBL;Acc:O97682] [ENSMMUT00000029735]	13.89	0.029
Macaca mulatta unknown Fragment [Source:UniProtKB/TrEMBL;Acc:O97682] [ENSMMUT00000029736]	12.35	0.046
succinate receptor 1 [Source:HGNC Symbol;Acc:4542] [ENSMMUT00000002359]	12.20	0.004
Macaca mulatta unknown Fragment [Source:UniProtKB/TrEMBL;Acc:O97682] [ENSMMUT00000029736]	12.20	0.041
Macaca mulatta indoleamine 2,3-dioxygenase 1 (IDO1), mRNA [NM_001077483]	10.10	0.003
Macaca mulatta indoleamine 2,3-dioxygenase 1 (IDO1), mRNA [NM_001077483]	10.00	0.008
annexin A3 [Source:HGNC Symbol;Acc:541] [ENSMMUT00000031250]	8.47	0.041
chemokine (C-X-C motif) ligand 6 (granulocyte chemotactic protein 2) [Source:HGNC Symbol;Acc:10643] [ENSMMUT00000025054]	8.00	0.042
inhibin, beta A [Source:HGNC Symbol;Acc:6066] [ENSMMUT00000019988]	7.58	0.037
Macaca mulatta tripartite motif-containing 5 (TRIM5), mRNA [NM_001032910]	7.52	0.048
EGF-like-domain, multiple 6 [Source:HGNC Symbol;Acc:3235] [ENSMMUT00000031824]	7.19	0.003
C-X-C motif chemokine 10 Precursor (Small-inducible cytokine B10)(10 kDa interferon-gamma-induced protein)(Gamma-IP10)(IP-10) [Source:UniProtKB/Swiss-Prot;Acc:Q8MIZ1] [ENSMMUT00000029391]	6.99	0.047
granzyme K (granzyme 3; tryptase II) [Source:HGNC Symbol;Acc:4711] [ENSMMUT00000028382]	6.13	0.002
Transferrin Fragment [Source:UniProtKB/TrEMBL;Acc:Q3YAS1] [ENSMMUT00000012466]	6.10	0.015
ST6 (alpha-N-acetyl-neuraminyl-2,3-beta-galactosyl-1,3)-N-acetylgalactosaminide alpha-2,6-sialyltransferase 2 [Source:HGNC Symbol;Acc:10867] [ENSMMUT00000031331]	5.92	0.024
ILLUMIGEN_MCQ_26220 Katze_MMBR Macaca mulatta cDNA clone IBIUW:8552 5' similar to Bases 3 to 1025 highly similar to human TF (Hs.433923), mRNA sequence [CN646444]	5.75	0.041
PREDICTED: Macaca mulatta retinoic acid receptor responder protein 3-like (LOC722189), mRNA [XM_001118373]	4.88	0.021
retinoic acid receptor responder (tazarotene induced) 3 [Source:HGNC Symbol;Acc:9869] [ENSMMUT00000025736]	4.72	0.011
Norrie disease (pseudoglioma) [Source:HGNC Symbol;Acc:7678] [ENSMMUT00000025286]	4.50	0.003
family with sequence similarity 26, member F [Source:HGNC Symbol;Acc:33391] [ENSMMUT00000010359]	4.48	0.012
ring finger protein 183 [Source:HGNC Symbol;Acc:28721] [ENSMMUT00000008196]	4.22	0.041
lipocalin 2 [Source:HGNC Symbol;Acc:6526] [ENSMMUT00000033493]	4.15	0.010
lipocalin 2 [Source:HGNC Symbol;Acc:6526] [ENSMMUT00000033493]	4.12	0.034
PREDICTED: Macaca mulatta tryptophanyl-tRNA synthetase, transcript variant 12 (WARS), mRNA [XM_001105926]	4.03	0.013
PREDICTED: Macaca mulatta ubiquitin [XR_012726]	4.00	0.025
caspase 1, apoptosis-related cysteine peptidase (interleukin 1, beta, convertase) [Source:HGNC Symbol;Acc:1499] [ENSMMUT00000013767]	3.80	0.021
ubiquitin-conjugating enzyme E2L 6 [Source:HGNC Symbol;Acc:12490] [ENSMMUT00000001886]	3.75	0.024
ephrin-A2 [Source:HGNC Symbol;Acc:3222] [ENSMMUT00000031095]	3.70	0.021
fibroblast activation protein, alpha [Source:HGNC Symbol;Acc:3590] [ENSMMUT00000004535]	3.57	0.041
apolipoprotein L, 2 [Source:HGNC Symbol;Acc:619] [ENSMMUT00000002165]	3.50	0.025
Macaca mulatta outer dense fiber of sperm tails 3B (ODF3B), mRNA [NM_001194710]	3.50	0.046
Macaca mulatta CD274 molecule (CD274), mRNA [NM_001083889]	3.41	0.005
sulfatase 1 [Source:HGNC Symbol;Acc:20391] [ENSMMUT00000032745]	3.34	0.040
Macaca mulatta proteasome (prosome, macropain) subunit, beta type, 9 (large multifunctional peptidase 2) (PSMB9), mRNA [NM_001194864]	3.31	0.037
Macaca mulatta integrin beta 1 mRNA, partial cds. [AY878076]	3.23	0.007

PREDICTED: Macaca mulatta protein ALO17-like (LOC718036), mRNA [XM_001110231]	3.19	0.010
Macaca mulatta proteasome (prosome, macropain) subunit, beta type, 9 (large multifunctional peptidase 2) (PSMB9), mRNA [NM_001194864]	3.13	0.029
prostate transmembrane protein, androgen induced 1 [Source:HGNC Symbol;Acc:14107] [ENSMMUT00000031785]	3.09	0.015
signal peptide, CUB domain, EGF-like 3 [Source:HGNC Symbol;Acc:13655] [ENSMMUT00000033135]	3.06	0.041
glutamic pyruvate transaminase (alanine aminotransferase) 2 [Source:HGNC Symbol;Acc:18062] [ENSMMUT00000004645]	2.93	0.026
olfactory receptor, family 51, subfamily E, member 1 [Source:HGNC Symbol;Acc:15194] [ENSMMUT00000020907]	2.79	0.036
Macaca mulatta chemokine (C-C motif) receptor 5 (CCR5), mRNA [NM_001042773]	2.74	0.025
signal transducer and activator of transcription 1, 91kDa [Source:HGNC Symbol;Acc:11362] [ENSMMUT00000007897]	2.73	0.046
ILLUMIGEN_MCQ_28473 Katze_MMPB Macaca mulatta cDNA clone IBIUW:7603 5' similar to Bases 149 to 409 highly similar to human APOL1 (Hs.114309), mRNA sequence [CN647435]	2.67	0.037
baculoviral IAP repeat-containing 3 [Source:HGNC Symbol;Acc:591] [ENSMMUT00000004807]	2.62	0.040
platelet-derived growth factor receptor-like [Source:HGNC Symbol;Acc:8805] [ENSMMUT00000001624]	2.51	0.039
G protein-coupled receptor 115 [Source:HGNC Symbol;Acc:19011] [ENSMMUT00000025228]	2.49	0.006
Sushi-repeat protein [Source:UniProtKB/TrEMBL;Acc:A3FEK9] [ENSMMUT00000011478]	2.48	0.034
F-box protein 6 [Source:HGNC Symbol;Acc:13585] [ENSMMUT00000047575]	2.44	0.016
Macaca mulatta chloride channel accessory 1 (CLCA1), mRNA [NM_001032912]	2.43	0.012
nuclear factor (erythroid-derived 2)-like 3 [Source:HGNC Symbol;Acc:7783] [ENSMMUT00000005121]	2.39	0.008
transmembrane protein 190 [Source:HGNC Symbol;Acc:29632] [ENSMMUT00000022343]	2.36	0.029
Protein HIDE1 Precursor [Source:UniProtKB/Swiss-Prot;Acc:A8MVS5] [ENSMMUT00000026680]	2.36	0.041
F-box protein 6 [Source:HGNC Symbol;Acc:13585] [ENSMMUT00000047575]	2.28	0.032
cell division cycle 25 homolog B (S. pombe) [Source:HGNC Symbol;Acc:1726] [ENSMMUT00000026604]	2.25	0.035
ILLUMIGEN_MCQ_69095 Katze_MMTE Macaca mulatta cDNA clone IBIUW:39364 5' similar to Bases 7 to 610 highly similar to human IFITM3 (Hs.374650), mRNA sequence [DV769955]	2.25	0.036
tumor necrosis factor receptor superfamily, member 9 [Source:HGNC Symbol;Acc:11924] [ENSMMUT00000023238]	2.25	0.046
GRIP and coiled-coil domain containing 2 [Source:HGNC Symbol;Acc:23218] [ENSMMUT00000032158]	2.15	0.045
solute carrier family 6 (neurotransmitter transporter, taurine), member 6 [Source:HGNC Symbol;Acc:11052] [ENSMMUT00000008890]	2.04	0.046
pim-3 oncogene [Source:HGNC Symbol;Acc:19310] [ENSMMUT00000003046]	2.03	0.046
ILLUMIGEN_MCQ_27395 Katze_MMBR Macaca mulatta cDNA clone IBIUW:8115 5' similar to Bases 1 to 600 highly similar to human DSCR6 (Hs.254560), mRNA sequence [CN646916]	1.98	0.047
purinergic receptor P2Y, G-protein coupled, 14 [Source:HGNC Symbol;Acc:16442] [ENSMMUT00000024237]	1.95	0.041
purinergic receptor P2Y, G-protein coupled, 14 [Source:HGNC Symbol;Acc:16442] [ENSMMUT00000024237]	1.93	0.046
ERO1-like (S. cerevisiae) [Source:HGNC Symbol;Acc:13280] [ENSMMUT00000039844]	1.92	0.046
tribbles homolog 3 (Drosophila) [Source:HGNC Symbol;Acc:16228] [ENSMMUT00000017657]	1.69	0.024
PREDICTED: Macaca mulatta mitochondrial inner membrane protease ATP23 homolog (LOC712932), mRNA [XM_001102089]	0.62	0.046
solute carrier family 16, member 10 (aromatic amino acid transporter) [Source:HGNC Symbol;Acc:17027] [ENSMMUT00000030306]	0.55	0.027
Macaca mulatta lymphocyte antigen 6 complex, locus G6C (LY6G6C), mRNA [NM_001194576]	0.55	0.008
acetyl-CoA acyltransferase 1 [Source:HGNC Symbol;Acc:82] [ENSMMUT00000001420]	0.54	0.041
PREDICTED: Macaca mulatta jerky homolog-like (mouse) (JRKL), mRNA [XM_001094262]	0.54	0.041
Macaca mulatta multimerin 2 (MMRN2), mRNA [NM_001193776]	0.53	0.024
membrane-spanning 4-domains, subfamily A, member 13 [Source:HGNC Symbol;Acc:16674] [ENSMMUT00000044282]	0.52	0.010
plexin domain containing 1 [Source:HGNC Symbol;Acc:20945] [ENSMMUT00000006939]	0.49	0.020
matrilin 2 [Source:HGNC Symbol;Acc:6908] [ENSMMUT00000028224]	0.48	0.046
PREDICTED: Macaca mulatta protein kinase, AMP-activated, alpha 2 catalytic subunit (PRKAA2), partial mRNA [XM_002801568]	0.47	0.010
leucine rich repeat transmembrane neuronal 4 [Source:HGNC Symbol;Acc:19411] [ENSMMUT00000043428]	0.46	0.020
ILLUMIGEN_MCQ_10841 Katze_MMPL2 Macaca mulatta cDNA clone IBIUW:9592 5' similar to Bases 1 to 695 highly similar to human PP2135 (Hs.132569), mRNA sequence [CN644776]	0.46	0.041
Macaca mulatta forkhead box C2 (MFH-1, mesenchyme forkhead 1) (FOXC2), mRNA [NM_001198708]	0.46	0.012
PREDICTED: Macaca mulatta keratin-associated protein 13-1-like (LOC712810), mRNA [XM_001101916]	0.45	0.012
klotho [Source:HGNC Symbol;Acc:6344] [ENSMMUT00000023968]	0.45	0.003
Transmembrane protein C20orf123 [Source:UniProtKB/Swiss-Prot;Acc:Q9BR26] [ENSMMUT00000023768]	0.45	0.012
laminin, alpha 3 [Source:HGNC Symbol;Acc:6483] [ENSMMUT00000048264]	0.45	0.041
advillin [Source:HGNC Symbol;Acc:14188] [ENSMMUT00000007486]	0.44	0.033
programmed cell death 4 (neoplastic transformation inhibitor) [Source:HGNC Symbol;Acc:8763] [ENSMMUT00000024185]	0.42	0.025
Macaca mulatta dehydrogenase/reductase (SDR family) member 4 (DHRS4), mRNA [NM_001194475]	0.42	0.049
PREDICTED: Macaca mulatta hypothetical LOC721380 (LOC721380), mRNA [XM_001117508]	0.41	0.041

phosphatidylinositol glycan anchor biosynthesis, class Z [Source:HGNC Symbol;Acc:30596] [ENSMMUT00000045206]	0.41	0.009
calcium/calmodulin-dependent protein kinase II beta [Source:HGNC Symbol;Acc:1461] [ENSMMUT00000003466]	0.39	0.026
protein kinase, AMP-activated, alpha 2 catalytic subunit [Source:HGNC Symbol;Acc:9377] [ENSMMUT00000014567]	0.38	0.021
CNKSR family member 3 [Source:HGNC Symbol;Acc:23034] [ENSMMUT00000002065]	0.38	0.035
PREDICTED: Macaca mulatta insulin-like growth factor binding protein 1, transcript variant 3 (IGFBP1), mRNA [XM_001085935]	0.38	0.027
ILLUMIGEN_MCQ_6817 Katze_MMPL2 Macaca mulatta cDNA clone IBIUW:4419 5' similar to Bases 1 to 1071 highly similar to human FBXO32 (Hs.403933), mRNA sequence [CN802472]	0.37	0.022
PREDICTED: Macaca mulatta 39S ribosomal protein L54, mitochondrial-like (LOC713878), mRNA [XM_001100840]	0.37	0.019
Macaca mulatta KIAA1324-like (KIAA1324L), mRNA [NM_001194030]	0.36	0.006
PREDICTED: Macaca mulatta peroxisomal biogenesis factor 11 alpha, transcript variant 1 (PEX11A), mRNA [XM_001093381]	0.35	0.045
Macaca mulatta dehydrogenase/reductase (SDR family) member 4 (DHRS4), mRNA [NM_001194475]	0.35	0.033
PREDICTED: Macaca mulatta hypothetical protein LOC100424638 (LOC100424638), mRNA [XM_002802655]	0.33	0.035
PREDICTED: Macaca mulatta melanoma associated antigen (mutated) 1-like 1, transcript variant 3 (MUM1L1), mRNA [XM_001092843]	0.31	0.014
BCL2-like 10 (apoptosis facilitator) [Source:HGNC Symbol;Acc:993] [ENSMMUT00000024430]	0.31	0.004
PREDICTED: Macaca mulatta cytochrome c oxidase subunit 6C-like (LOC100423334), mRNA [XM_002802732]	0.30	0.033
protease, serine, 42 [Source:HGNC Symbol;Acc:30716] [ENSMMUT00000020676]	0.30	0.003
PREDICTED: Macaca mulatta ectodysplasin A, transcript variant 1 (EDA), mRNA [XM_001082424]	0.29	0.031
PREDICTED: Macaca mulatta sema domain, immunoglobulin domain (Ig), short basic domain, secreted, (semaphorin) 3B, transcript variant 1 (SEMA3B), mRNA [XM_001102773]	0.28	0.033
HHIP-like 2 [Source:HGNC Symbol;Acc:25842] [ENSMMUT00000011055]	0.27	0.023
phospholipid scramblase 4 [Source:HGNC Symbol;Acc:16497] [ENSMMUT00000045531]	0.27	0.005
leucine rich repeat transmembrane neuronal 4 [Source:HGNC Symbol;Acc:19411] [ENSMMUT00000043428]	0.26	0.002
PREDICTED: Macaca mulatta SH3-domain GRB2-like 2 (SH3GL2), mRNA [XM_001102732]	0.25	0.010
calcium/calmodulin-dependent protein kinase II beta [Source:HGNC Symbol;Acc:1461] [ENSMMUT00000003462]	0.25	0.039
Xg pseudogene, Y-linked 2 [Source:HGNC Symbol;Acc:34022] [ENSMMUT00000048240]	0.25	0.041
Macaca mulatta neuropeptide Y receptor Y5 (NPY5R), mRNA [NM_001032833]	0.25	0.010
PREDICTED: Macaca mulatta hemogen, transcript variant 4 (HEMGN), mRNA [XM_001113799]	0.24	0.041
Uncharacterized protein C1orf115 [Source:UniProtKB/Swiss-Prot;Acc:Q9H7X2] [ENSMMUT00000033027]	0.23	0.008
dynein, axonemal, heavy chain 7 [Source:HGNC Symbol;Acc:18661] [ENSMMUT00000007911]	0.23	0.040
Macaca mulatta neuropeptide Y receptor Y1 (NPY1R), mRNA [NM_001032866]	0.23	0.002
homeobox D1 [Source:HGNC Symbol;Acc:5132] [ENSMMUT00000031334]	0.23	0.024
ATP-binding cassette, sub-family A (ABC1), member 8 [Source:HGNC Symbol;Acc:38] [ENSMMUT00000026133]	0.22	0.012
histidine rich carboxyl terminus 1 [Source:HGNC Symbol;Acc:33872] [ENSMMUT00000022949]	0.22	0.046
Dynein Fragment [Source:UniProtKB/TrEMBL;Acc:Q6UIQ6] [ENSMMUT00000008273]	0.20	0.002
POU class 2 homeobox 3 [Source:HGNC Symbol;Acc:19864] [ENSMMUT00000017160]	0.20	0.008
RUN domain containing 3B [Source:HGNC Symbol;Acc:30286] [ENSMMUT00000009468]	0.20	0.047
protein serine kinase H2 [Source:HGNC Symbol;Acc:18997] [ENSMMUT00000002287]	0.20	0.024
neuropeptide Y receptor Y1 [Source:RefSeq peptide;Acc:NP_001028038] [ENSMMUT00000028477]	0.19	0.002
PREDICTED: Macaca mulatta putative peptide YY-3-like (LOC694766), mRNA [XM_001083655]	0.19	0.024
Macaca mulatta myosin, heavy chain 2, skeletal muscle, adult (MYH2), mRNA [NM_001195292]	0.18	0.041
Macaca mulatta myosin, heavy chain 2, skeletal muscle, adult (MYH2), mRNA [NM_001195292]	0.18	0.010
PREDICTED: Macaca mulatta keratin associated protein 13-2 (KRTAP13-2), mRNA [XM_001099939]	0.17	0.004
metallothionein 1B [Source:HGNC Symbol;Acc:7394] [ENSMMUT00000039322]	0.14	0.037
Dynein Fragment [Source:UniProtKB/TrEMBL;Acc:Q6UIQ6] [ENSMMUT00000008273]	0.14	0.003
retinol binding protein 4, plasma [Source:HGNC Symbol;Acc:9922] [ENSMMUT00000020627]	0.14	0.012
PREDICTED: Macaca mulatta hypothetical LOC701270 (LOC701270), mRNA [XM_001089592]	0.14	0.015
MAM domain containing 2 [Source:HGNC Symbol;Acc:23673] [ENSMMUT00000011666]	0.14	0.002
retinol binding protein 4, plasma [Source:HGNC Symbol;Acc:9922] [ENSMMUT00000020627]	0.14	0.015
SVOP-like [Source:HGNC Symbol;Acc:27034] [ENSMMUT00000030500]	0.12	0.024
metallothionein 1B [Source:HGNC Symbol;Acc:7394] [ENSMMUT00000039324]	0.08	0.006
immunoglobin superfamily, member 21 [Source:HGNC Symbol;Acc:28246] [ENSMMUT00000033081]	0.08	0.046
dehydrogenase/reductase (SDR family) member 7C [Source:HGNC Symbol;Acc:32423] [ENSMMUT00000042221]	0.06	0.002
Uncharacterized protein C11orf16 [Source:UniProtKB/Swiss-Prot;Acc:Q9NQ32] [ENSMMUT00000002826]	0.06	0.010
cytochrome P450, family 3, subfamily A, polypeptide 5 [Source:RefSeq peptide;Acc:NP_001035309] [ENSMMUT00000005022]	0.06	0.015
