# Supplementary material for: Therapeutic Helminth Infection of Macaques with Idiopathic Chronic Diarrhea Alters the Inflammatory Signature and Mucosal Microbiota of the Colon
Source: PLoS Pathog. 2012 Nov 15;8(11):e1003000. doi: 10.1371/journal.ppat.1003000 (PMC3499566; doi:10.1371/journal.ppat.1003000)
Supplement: Table S2 — List of genes differentially expressed in colon biopsies from colitis subjects before and after T. trichiura treatment. Genes most highly expressed following treatment are listed at the top. FDR, false discovery rate. (PDF) [file ppat.1003000.s009.pdf]

Table S2

Description	Fold	FDR
acyl-CoA synthetase bubblegum family member 1 [Source:HGNC Symbol;Acc:29567] [ENSMMUT00000016586]	6.00	0.174
interleukin 9 receptor [Source:HGNC Symbol;Acc:6030] [ENSMMUT00000002856]	5.36	0.280
PREDICTED: Macaca mulatta arachidonate 5-lipoxygenase, transcript variant 1 (ALOX5), mRNA [XM_001102354]	5.13	0.174
arachidonate 15-lipoxygenase [Source:HGNC Symbol;Acc:433] [ENSMMUT00000042388]	5.13	0.174
carboxypeptidase A3 (mast cell) [Source:HGNC Symbol;Acc:2298] [ENSMMUT00000007772]	4.85	0.273
membrane-spanning 4-domains, subfamily A, member 2 (Fc fragment of IgE, high affinity I, receptor for; beta polypeptide) [Source:HGNC Symbol;Acc:7316] [ENSMMUT00000018994]	4.71	0.174
arachidonate 5-lipoxygenase [Source:HGNC Symbol;Acc:435] [ENSMMUT00000018848]	4.68	0.202
Putative uncharacterized protein C1orf150 [Source:UniProtKB/Swiss-Prot;Acc:Q5JQS6] [ENSMMUT00000044542]	4.62	0.211
carboxypeptidase A3 (mast cell) [Source:HGNC Symbol;Acc:2298] [ENSMMUT00000007772]	4.49	0.175
PREDICTED: Macaca mulatta mastin-like (LOC722431), mRNA [XM_001118581]	4.46	0.174
tenascin C [Source:HGNC Symbol;Acc:5318] [ENSMMUT00000004742]	4.38	0.174
baculoviral IAP repeat-containing 7 [Source:HGNC Symbol;Acc:13702] [ENSMMUT00000012902]	3.55	0.174
leukotriene C4 synthase [Source:HGNC Symbol;Acc:6719] [ENSMMUT00000017351]	3.53	0.174
acyl-CoA synthetase bubblegum family member 1 [Source:HGNC Symbol;Acc:29567] [ENSMMUT00000016586]	3.45	0.174
tenascin C [Source:HGNC Symbol;Acc:5318] [ENSMMUT00000004742]	3.40	0.288
PREDICTED: Macaca mulatta dermatopontin-like (LOC700181), mRNA [XM_002802062]	3.36	0.273
ghrelin [Source:RefSeq peptide;Acc:NP_001028075] [ENSMMUT00000028003]	3.27	0.213
G protein-coupled receptor 44 [Source:HGNC Symbol;Acc:4502] [ENSMMUT00000000025]	3.14	0.248
Fibronectin 1 Fragment [Source:UniProtKB/TrEMBL;Acc:Q5MD86] [ENSMMUT00000017323]	2.98	0.174
Fibronectin 1 Fragment [Source:UniProtKB/TrEMBL;Acc:Q5MD86] [ENSMMUT00000017323]	2.96	0.174
PREDICTED: Macaca mulatta collagen alpha-1(XIII) chain-like (LOC716986), mRNA [XM_001108092]	2.95	0.175
arachidonate 5-lipoxygenase [Source:HGNC Symbol;Acc:435] [ENSMMUT00000018848]	2.94	0.174
PREDICTED: Macaca mulatta mastin-like (LOC722427), mRNA [XM_001118577]	2.88	0.174
dermatopontin [Source:HGNC Symbol;Acc:3011] [ENSMMUT00000004157]	2.72	0.174
PREDICTED: Macaca mulatta protein FAM38A-like (LOC722206), mRNA [XM_002800862]	2.59	0.174
myosin binding protein H [Source:HGNC Symbol;Acc:7552] [ENSMMUT00000012176]	2.54	0.279
prostaglandin-endoperoxide synthase 1 (prostaglandin G/H synthase and cyclooxygenase) [Source:HGNC Symbol;Acc:9604] [ENSMMUT00000018079]	2.50	0.273
insulin-like growth factor binding protein 6 [Source:HGNC Symbol;Acc:5475] [ENSMMUT00000028608]	2.42	0.174
PREDICTED: Macaca mulatta collagen, type III, alpha 1 (COL3A1), mRNA [XM_001105968]	2.39	0.260
CD320 molecule [Source:HGNC Symbol;Acc:16692] [ENSMMUT00000023457]	2.33	0.246
v-kit Hardy-Zuckerman 4 feline sarcoma viral oncogene homolog [Source:HGNC Symbol;Acc:6342] [ENSMMUT00000004330]	2.17	0.174
ILLUMIGEN_MCQ_32942 Katze_MMBR Macaca mulatta cDNA clone IBIUW:12912 5' similar to Bases 1 to 238 highly similar to human IGFBP6 (Hs.274313), mRNA sequence [CN803406]	2.09	0.175
PREDICTED: Macaca mulatta cysteine-rich secretory protein LCCL domain containing 2, transcript variant 3 (CRISPLD2), mRNA [XM_001112988]	2.02	0.193
PREDICTED: Macaca mulatta prostaglandin-endoperoxide synthase 1, transcript variant 6 (PTGS1), mRNA [XM_001088270]	2.01	0.280
Macaca mulatta collagen type III alpha 1 mRNA, 3' UTR. [FJ932748]	1.98	0.273
spondin 1, extracellular matrix protein [Source:HGNC Symbol;Acc:11252] [ENSMMUT00000030829]	1.83	0.288
early B-cell factor 4 [Source:HGNC Symbol;Acc:29278] [ENSMMUT00000044592]	1.82	0.174
ArfGAP with GTPase domain, ankyrin repeat and PH domain 1 [Source:HGNC Symbol;Acc:16922] [ENSMMUT00000013063]	1.80	0.174
zinc finger protein 620 [Source:HGNC Symbol;Acc:28742] [ENSMMUT00000022433]	1.76	0.279
Macaca mulatta carbonyl reductase 3 (CBR3), mRNA [NM_001193746]	1.68	0.267
insulin-like growth factor 1 receptor [Source:HGNC Symbol;Acc:5465] [ENSMMUT00000017236]	1.43	0.282
F-box protein 6 [Source:HGNC Symbol;Acc:13585] [ENSMMUT00000047575]	0.65	0.273
Macaca mulatta haloacid dehalogenase-like hydrolase domain containing 3 (HDHD3), mRNA [NM_001194198]	0.56	0.271
signal transducer and activator of transcription 1, 91kDa [Source:HGNC Symbol;Acc:11362] [ENSMMUT00000007897]	0.51	0.174
ubiquitin-conjugating enzyme E2L 6 [Source:HGNC Symbol;Acc:12490] [ENSMMUT00000001886]	0.49	0.174
CD5 molecule-like [Source:HGNC Symbol;Acc:1690] [ENSMMUT00000027876]	0.48	0.287
PREDICTED: Macaca mulatta ubiquitin [XR_012726]	0.47	0.175
retinoic acid receptor responder (tazarotene induced) 3 [Source:HGNC Symbol;Acc:9869] [ENSMMUT00000025736]	0.45	0.280
PREDICTED: Macaca mulatta thymidine phosphorylase, transcript variant 4 (TYMP), mRNA [XM_001112945]	0.45	0.288
G protein-coupled receptor 128 [Source:HGNC Symbol;Acc:19241] [ENSMMUT00000004053]	0.43	0.174
PREDICTED: Macaca mulatta retinoic acid receptor responder protein 3-like (LOC722189), mRNA [XM_001118373]	0.41	0.174
PREDICTED: Macaca mulatta hypothetical LOC703547, transcript variant 1 (LOC703547), mRNA [XM_001099113]	0.40	0.288
PREDICTED: Macaca mulatta hypothetical LOC703547, transcript variant 1 (LOC703547), mRNA [XM_001099113]	0.40	0.288
PREDICTED: Macaca mulatta guanylate-binding protein 6-like (LOC695158), miscRNA [XR_091839]	0.39	0.174
CD8a molecule [Source:HGNC Symbol;Acc:1706] [ENSMMUT00000004986]	0.39	0.288
Macaca mulatta indoleamine 2,3-dioxygenase 1 (IDO1), mRNA [NM_001077483]	0.39	0.174
Macaca mulatta indoleamine 2,3-dioxygenase 1 (IDO1), mRNA [NM_001077483]	0.38	0.273
tubulointerstitial nephritis antigen [Source:HGNC Symbol;Acc:14599] [ENSMMUT00000017935]	0.32	0.174
sciellin [Source:HGNC Symbol;Acc:10573] [ENSMMUT00000000066]	0.30	0.282
PREDICTED: Macaca mulatta thymidine phosphorylase, transcript variant 4 (TYMP), mRNA [XM_001112945]	0.29	0.288
ring finger protein 182 [Source:HGNC Symbol;Acc:28522] [ENSMMUT00000041378]	0.27	0.202
C-X-C motif chemokine 10 Precursor (Small-inducible cytokine B10)(10 kDa interferon-gamma-induced protein)(Gamma-IP10)(IP-10) [Source:UniProtKB/Swiss-Prot;Acc:Q8MIZ1] [ENSMMUT00000029391]	0.27	0.174
Macaca mulatta chemokine (C-X-C motif) ligand 10 (CXCL10), mRNA [NM_001032892]	0.24	0.174
tumor protein p63 regulated 1 [Source:HGNC Symbol;Acc:24759] [ENSMMUT00000009593]	0.23	0.174
PREDICTED: Macaca mulatta hephaestin-like 1 (HEPHL1), mRNA [XM_001086917]	0.22	0.175
Macaca mulatta chemokine (C-X-C motif) ligand 11 (CXCL11), mRNA [NM_001032950]	0.19	0.174
small inducible cytokine B11 [Source:RefSeq peptide;Acc:NP_001028122] [ENSMMUT00000029393]	0.16	0.174
