# Supplementary material for: Therapeutic Helminth Infection of Macaques with Idiopathic Chronic Diarrhea Alters the Inflammatory Signature and Mucosal Microbiota of the Colon
Source: PLoS Pathog. 2012 Nov 15;8(11):e1003000. doi: 10.1371/journal.ppat.1003000 (PMC3499566; doi:10.1371/journal.ppat.1003000)
Supplement: Table S3 — List of genes differentially expressed in colon biopsies between clinical responders (R) and subject TC05 (NR) pre-treatment with T. trichiura . Genes most highly expressed in the non-responding subject TC05 are listed at the top. FDR, false discovery rate. (PDF) [file ppat.1003000.s010.pdf]

Table S3

ProbeID	Description	Fold	FDR
A_01_P1692126		38.239	0.04064
A_01_P1819261		37.096	0.00087
A_01_P1703526		18.885	0.00781
A_01_P1802881		11.94	0.04064
A_01_P1832812		10.216	0.01363
A_01_P1793846		6.044	0.01301
A_01_P006328	sphingosine-1-phosphate phosphatase 2 [Source:HGNC Symbol;Acc:19953] [ENSMMUT00000010762]	4.448	0.02617
A_01_P012199	paraoxonase 3 [Source:HGNC Symbol;Acc:9206] [ENSMMUT00000011208]	0.068	0.04831
A_01_P1825581	PREDICTED: Macaca mulatta 40S ribosomal protein S27-like (LOC696068), mRNA [XM_001084710]	0.061	0.00242
A_01_P020134	PREDICTED: Macaca mulatta mediator complex subunit 17, transcript variant 4 (MED17), mRNA [XM_001088365]	0.05	0.00474
A_01_P1696961	Macaca mulatta clone 464LK1 immunoglobulin kappa light chain (IGK) mRNA, partial cds. [FJ795842]	0.018	0.00711
A_01_P1736386		0.016	0.00058
A_01_P1736376		0.014	0.01937
A_01_P1694966	immunoglobulin kappa constant [Source:HGNC Symbol;Acc:5716] [ENSMMUT00000029842]	0.012	0.00058
A_01_P1682792	immunoglobulin kappa constant [Source:HGNC Symbol;Acc:5716] [ENSMMUT00000029842]	0.011	0.00058
A_01_P1736936		0.011	0.01039
A_01_P1697811	Macaca mulatta Ig rearranged light chain variable region, anti-RBC antibody, mRNA, partial cds. [U57571]	0.011	0.00347
A_01_P1699936		0.01	0.01247
A_01_P1696840	immunoglobulin kappa constant [Source:HGNC Symbol;Acc:5716] [ENSMMUT00000029842]	0.009	0.00079
A_01_P1736906	PREDICTED: Macaca mulatta hypothetical LOC707609 (LOC707609), mRNA [XM_001096027]	0.008	0.04064
A_01_P1697816	Macaca mulatta Ig rearranged light chain variable region, anti-RBC antibody, mRNA, partial cds. [U57572]	0.008	0.00908
A_01_P1696981	Macaca mulatta clone 464LK8 immunoglobulin kappa light chain (IGK) mRNA, partial cds. [FJ795847]	0.007	0.0193
A_01_P1696176	immunoglobulin kappa constant [Source:HGNC Symbol;Acc:5716] [ENSMMUT00000029842]	0.007	0.00058
A_01_P1736381	immunoglobulin kappa constant [Source:HGNC Symbol;Acc:5716] [ENSMMUT00000029842]	0.007	0.00058
A_01_P1696856	immunoglobulin kappa constant [Source:HGNC Symbol;Acc:5716] [ENSMMUT00000029842]	0.007	0.00087
A_01_P1682796	Macaca mulatta Ig rearranged light chain variable region, anti-RBC antibody, mRNA, partial cds. [U57570]	0.006	0.00079
A_01_P1696181	Macaca mulatta clone D0(8) immunoglobulin light chain mRNA, partial cds. [AY994523]	0.006	0.00058

A_01_P1698851	Macaca mulatta clone D0(2) immunoglobulin light chain mRNA, partial cds. [AY994517]	0.006	0.00058
A_01_P1682936	Macaca mulatta Ig rearranged light chain variable region, anti-RBC antibody, mRNA, partial cds. [U57575]	0.006	0.01238
A_01_P1701676	Macaca mulatta clone S19 immunoglobulin kappa light chain mRNA, partial cds. [AY452636]	0.006	0.00058
A_01_P1701661	Macaca mulatta Ig rearranged light chain variable region, anti-RBC antibody, mRNA, partial cds. [U57570]	0.006	0.00064
A_01_P1736396		0.006	0.02009
A_01_P1696186	Macaca mulatta clone D0(15) immunoglobulin light chain mRNA, partial cds. [AY994524]	0.006	0.00058
A_01_P1701671		0.006	0.00434
A_01_P1736921		0.006	0.00938
A_01_P1697821	Macaca mulatta Ig rearranged light chain variable region, anti-RBC antibody, mRNA, partial cds. [U57574]	0.005	0.00288
A_01_P1696196	Macaca mulatta clone D21(15) immunoglobulin light chain mRNA, partial cds. [AY994526]	0.005	0.00058
A_01_P1736366	PREDICTED: Macaca mulatta hypothetical protein LOC100424267 (LOC100424267), mRNA [XM_002799331]	0.005	0.00087
A_01_P1736391		0.005	0.00079
A_01_P1736896		0.005	0.01301
A_01_P1682931	Macaca mulatta Ig rearranged light chain variable region, anti-RBC antibody, mRNA, partial cds. [U57579]	0.005	0.00058
A_01_P1818496		0.005	0.01301
A_01_P1672341		0.005	0.0012
A_01_P1695881	Macaca mulatta clone 4S7K2_S22 immunoglobulin kappa chain (IgK) mRNA, partial cds. [AY452596]	0.004	0.03106
A_01_P1682926	Macaca mulatta clone B417K immunoglobulin kappa light chain (IGK) mRNA, partial cds. [HM044989]	0.004	0.02009
A_01_P1736881	Macaca mulatta clone B442K immunoglobulin kappa light chain (IGK) mRNA, partial cds. [HM044994]	0.004	0.01258
A_01_P1736916	Macaca mulatta clone B408K immunoglobulin kappa light chain (IGK) mRNA, partial cds. [HM044987]	0.004	0.02142
